# Supplementary material for: BmAbl1 Regulates Silk Protein Synthesis via Glutathione Metabolism in Bombyx mori
Source: Insects. 2022 Oct 22;13(11):967. doi: 10.3390/insects13110967 (PMC9696079; doi:10.3390/insects13110967)

File S1. Detection of knockout sites at mRNA and protein level in transgenic silkworm

mRNA:

Nistari AAGCCACGGGTGCCGAAGGACCCCCGCATGCTCGGCTCCAACATATTCACTGAACATAGCGAGGCGCTGCTGCAGAGCCGA 153
BmAbl^{-} AAGCCACGGGTGCCGAAGGACCCCCGCATGCTCGGCTCCAACATATTCACTGAACATAGCG-- 133

Nistari CCGCTGCCGCACATCCCAGACCTGCCCGACGAGGCCGCCGCGCCACCCGCTCCGCTGCCGCTCGACTCCGCTAACAGATG 233
BmAbl^{-} -- 133

Nistari GACCTCGAAGGAGAATCTTTTGGCCCATCACGAAGAGGACGATCCCCAGCTGTTCGTAGCCCTCTACGATTTCCAGGCGGGA 315
BmAbl^{-} -- 133

Nistari GGGGAGAACCAGCTCACGCTCAAGAAAGGCGAGCAGGTGCGCATCATGAGCTACAACAAGAGCGGCGAGTGGTGCGAAGC 395
BmAbl^{-} ---AGGCGAGCAGGTGCGCATCATGAGCTACAACAAGAGCGGCGAGTGGTGCGAAGC 187

Nistari CCACACGCTGACTGGGGCCGTGGGCTGGGTGCCCAGCAACTACGTCACTCCAGTCAACAGTCTCGAGAAGCATTCATGGTA 476
BmAbl^{-} CCACACGCTGACTGGGGCCGTGGGCTGGGTGCCCAGCAACTACGTCACTCCAGTCAACAGTCTCGAGAAGCATTCATGGTA 268

Nistari CCATGGGCCGATTTCCCGCAACGCGGCGGAGTACCTCCTCTCGTCTGGGATCAATGGCAGCTTCCTGGTCCGCGAATCGGA 557
BmAbl^{-} CCATGGGCCGATTTCCCGCAACGCGGCGGAGTACCTCCTCTCGTCTGGGATCAATGGCAGCTTCCTGGTCCGCGAATCGGA 349

Nistari ATCGAGTCCTGGGCAACGCAGCATATCCCTCCGCTACGAAGGGAGAGTTTACCACTACCGCATCAACGAAGACGCCGATGG 638
BmAbl^{-} ATCGAGTCCTGGGCAACGCAGCATATCCCTCCGCTACGAAGGGAGAGTTTACCACTACCGCATCAACGAAGACGCCGATGG 430

Nistari GAAGGTT (573bp) 645
BmAbl^{-} GAAGGTT (-208bp) 437

Protein:

Nistari KPRVPKDPRMLGSNIFTEHSEALLQSRPLPHIPDLPDEAAAPPAPLPLDSANRWTSKENLLAHHEEDDPQLFVALYDFQAGG
BmAbl^{-} KPRVPKDPRMLGSNIFTEHSEASRCAS*--

Nistari ENQLT LKKGEQVRIMSYNKSGEWCEAHTLTGAVGWVPSNYVTPVNSLEKHSWYHGPISRNAAEYLLSSGINGSFLVRESES

Nistari SPGQRSISLRYEGRVYHYRINEDADGKVKPRV (191aa)
BmAbl^{-} --- (early termination)

BmAbl1 qRT-PCR：

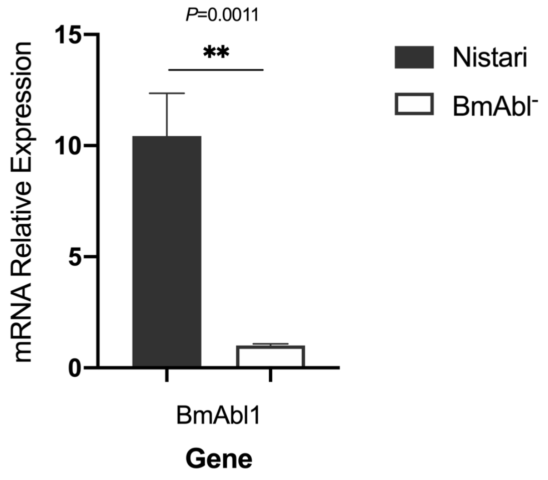

Supplement: Supplementary file 1 [file insects-13-00967-s001.zip › File S1.pdf]
