# Supplementary material for: BmAbl1 Regulates Silk Protein Synthesis via Glutathione Metabolism in Bombyx mori
Source: Insects. 2022 Oct 22;13(11):967. doi: 10.3390/insects13110967 (PMC9696079; doi:10.3390/insects13110967)
Supplement: Supplementary file 1 [file insects-13-00967-s001.zip › Table S1.pdf]

Table S1. The primers used to qRT-PCR.

Gene		Sequence (5'-3')	Efficiency
BMSK0001189	F	GCCGTTCACAGCCAAGAACC	106%
	R	CCACCGGGGACTTTTCAGTTTG	
BMSK0001495	F	AAATCCTTGACGCAGCCGGT	105%
	R	CTCCCATTCTCCCCTGCGTA	
BMSK0003392	F	ACTGCTTGTGGACCTGCTTTTATTT	105%
	R	AGTGGAACCAGCAGGAGAACA	
BMSK0005067	F	GAGCACGGAATGAAGGTAGGAGT	102%
	R	GTTGGCTCCTGCATTGGCAC	
BMSK0005586	F	CACTGACACCCGCCTTTGGA	102%
	R	AGCCCATGCGTGACTCAACAA	
BMSK0007210	F	ACTTTTTCGTGTTACGACGCTTCT	94%
	R	CGGTGGATATGTCCAACTGTTTTCG	
BMSK0009961	F	TTGGACCGGCGCACATACT	102%
	R	ACCTCTGTCTGAAACAGCCCC	
BMSK0001869	F	TGCAAGATGGCAAGGTGTCC	98%
	R	CTGGCTTCTTTGTAGCGTGC	
BMSK0003439	F	AAGAGCGGAAAAATGCCCGT	95%
	R	ATAACAGCACGACAAGGCGG	
BMSK0003598	F	CAGCGAAGGCGCTCAACTTA	103%
	R	AGCATCAAAACCATTCGTGACTTA	
BMSK0003984	F	TGATTTGGGACAGCCACGC	103%
	R	ACGCTCTTTTCTTGGGCTCG	
BMSK0006071	F	CAAGAATCGGTCTCCACGCC	100%
	R	GGGGTTTGTGCCGGTCTATG	
BMSK0006405	F	TGTTGCTGCGTACCACAAGG	98%
	R	TCCAGGTTCACTTCCGTCCA	
BMSK0012237	F	TACGCCAACAAGACACACCG	106%
	R	TAGTGCCCTTTTACTCTGTATCCTT	
Fib-L	F	ATACCGATTGGTCACATAACAG	105%

	R	GCAGATAGATGGGCGATAA	
Fib-H	F	TCTGTGTCATCTGCTTCATCTCG	100%
	R	TATCCAGGACGAAGTAAGAAACAA	
p25	F	AGCCGCTGTGGCAGTTTTG	97%
	R	TAGGTGGCGTTGAAGTATG	
Ser1	F	GATTGCGTTGGCTGCGCT	102%
	R	CCGTTCGGATCGCTGTGATA	
BmAbl	F	TCAAGGACGATACGATGGCGC	101%
	R	GTGTTGTAGGCCAGACCCTCC	
