# Supplementary material for: BmAbl1 Regulates Silk Protein Synthesis via Glutathione Metabolism in Bombyx mori
Source: Insects. 2022 Oct 22;13(11):967. doi: 10.3390/insects13110967 (PMC9696079; doi:10.3390/insects13110967)
Supplement: Supplementary file 1 [file insects-13-00967-s001.zip › Table S2.pdf]

Table S2. The primers used to construct pXL-[RFP-U6-BmAbl1-sgRNA].

**Primer**	**Sequence (5'-3')**
Abl1-sgRNA1.F	GGCGTCGACGGTGGGCGCGGCGGCCTCGTGTTTTAGAGCTAGAAATAGCAAGT
sgRNA1.R	ATGGTCGACAAAAAAAGCACCGACTCGGTGCCA
Abl1-sgRNA2.F	GGCGTCGACGGGGATCGTCCTCTTCGTGAGTTTTAGAGCTAGAAATAGCAAGT
sgRNA2.R	ATGGCTAGCAAAAAAAGCACCGACTCGGTGCCA
