# Supplementary material for: BmAbl1 Regulates Silk Protein Synthesis via Glutathione Metabolism in Bombyx mori
Source: Insects. 2022 Oct 22;13(11):967. doi: 10.3390/insects13110967 (PMC9696079; doi:10.3390/insects13110967)
Supplement: Supplementary file 1 [file insects-13-00967-s001.zip › Table S3.pdf]

Table S3. The primers used to detect mutagenesis.

Primer	Sequence (5'-3')
Abl1-gDNA-knock-out.F	TGACTTAGAGAGACGTACTGTTCA
Abl1-gDNA-knock-out.R	GCTGATGTCCATTGTGTCGC
Abl1-mRNA-knock-out.F	AAGCCACGGGTGCCGAA
Abl1-mRNA-knock-out.R	AACCTTCCCATCGGCGTCTT
