# Supplementary material for: BmAbl1 Regulates Silk Protein Synthesis via Glutathione Metabolism in Bombyx mori
Source: Insects. 2022 Oct 22;13(11):967. doi: 10.3390/insects13110967 (PMC9696079; doi:10.3390/insects13110967)
Supplement: Supplementary file 1 [file insects-13-00967-s001.zip › Table S4.pdf]

Table S4 Differentially expressed genes between Bmabl1- and Nistari

GeneID	Abl_1	Abl_2	Abl_3	Nis_1	Nis_2	Nis_3	baseMean	log2FoldChange	lfcSE	stat	pvalue	padj
BMSK0012305	2369	1247	3244	0	0	0	1070.18411	13.44593423	1.29956891	10.34645729	4.34E-25	8.43E-23
BMSK0008460	665	135	290	0	0	0	156.684831	10.67674666	1.24298109	8.589629218	8.73E-18	9.71E-16
BMSK0012306	131	41	195	0	0	0	56.642558	9.206637752	1.29295874	7.120596693	1.07E-12	8.38E-11
BMSK0011196	129	72	73	0	0	0	41.6493084	8.764166584	1.26704642	6.917005144	4.61E-12	3.31E-10
BMSK0007503	101	52	70	0	0	0	33.9570709	8.469591351	1.28331783	6.599761276	4.12E-11	2.63E-09
BMSK0000814	17	91	78	0	0	0	31.9848564	8.376104372	1.37016386	6.113213617	9.76E-10	4.98E-08
BMSK0001583	1800	818	1135	2	6	2	567.905431	8.365570499	0.51287084	16.31126164	8.21E-60	6.24E-57
BMSK0012909	63	35	51	0	0	0	22.8842858	7.899491522	1.32556476	5.959340329	2.53E-09	1.24E-07
BMSK0009847	50	28	49	0	0	0	19.6325152	7.677829059	1.35034682	5.68582007	1.30E-08	5.71E-07
BMSK0014042	44	28	37	0	0	0	16.8698311	7.458779197	1.3692279	5.447434435	5.11E-08	2.01E-06
BMSK0016060	56	36	11	0	0	0	15.4854342	7.337184947	1.42853586	5.136157342	2.80E-07	9.65E-06
BMSK0011560	644	996	1059	6	12	0	444.738676	7.203732645	1.00598753	7.160856772	8.02E-13	6.31E-11
BMSK0012819	490	719	847	9	0	4	338.361982	7.113495775	1.08390413	6.562845913	5.28E-11	3.33E-09
BMSK0000898	60	2	16	0	0	0	10.5552003	6.791982283	1.6038682	4.234750883	2.29E-05	0.00050788
BMSK0006932	26	18	15	0	0	0	9.08572072	6.56628917	1.50900822	4.351393918	1.35E-05	0.00032055
BMSK0010212	12	16	20	0	0	0	7.80467057	6.341926703	1.56946964	4.04080877	5.33E-05	0.00107751
BMSK0001332	17	10	16	0	0	0	6.65287831	6.116517742	1.61170591	3.795058204	0.00014761	0.00259467
BMSK0010659	21	7	16	0	0	0	6.59995704	6.108264458	1.62605407	3.7564953	0.00017231	0.00295485
BMSK0001483	19	8	14	0	0	0	6.19883511	6.01694488	1.64154782	3.665409448	0.00024694	0.00408127
BMSK0002432	404	235	207	2	2	8	130.689449	5.946378691	0.52583313	11.30848997	1.19E-29	3.20E-27
BMSK0000953	76	30	34	1	0	1	21.1120629	5.892095175	1.19600344	4.926486814	8.37E-07	2.60E-05
BMSK0001608	57	62	68	0	0	3	30.5008768	5.838466868	1.00112405	5.83191151	5.48E-09	2.55E-07
BMSK0012908	15	9	10	0	0	0	5.21221081	5.765207123	1.70707035	3.377252224	0.00073214	0.01022864
BMSK0010933	12	6	15	0	0	0	5.12238534	5.739247632	1.73008677	3.317317801	0.00090886	0.01217554
BMSK0003512	32	2	4	0	0	0	5.0413059	5.729670572	1.84642577	3.103114488	0.00191496	0.02217023
BMSK0005259	5	15	10	0	0	0	5.06794333	5.715301897	1.76260939	3.242523237	0.00118476	0.01501704
BMSK0009664	22	4	8	0	0	0	4.83030136	5.663204998	1.77872684	3.183853127	0.00145329	0.01774878
BMSK0001582	14	10	6	0	0	0	4.60075155	5.584837015	1.77335825	3.149299928	0.00163662	0.01941599
BMSK0013841	10	10	8	0	0	0	4.43592385	5.528952294	1.78541885	3.096725618	0.00195671	0.02252266
BMSK0015893	20	1	8	0	0	0	4.02705003	5.402386242	1.93507625	2.791820854	0.00524124	0.04919321
BMSK0015706	17	22	6	1	0	0	7.38400945	5.262383749	1.59103744	3.307517239	0.00094127	0.01251074
BMSK0001495	218	144	152	3	10	0	81.3866113	5.176366622	0.55778239	9.280261909	1.69E-20	2.37E-18
BMSK0015459	18	30	27	0	0	2	12.6687429	5.133293008	1.31365235	3.907649542	9.32E-05	0.00173875
BMSK0001840	38	17	16	0	0	2	10.9282395	4.922071815	1.33821691	3.678082203	0.00023499	0.00391214
BMSK0000815	62	176	112	10	2	0	61.2411022	4.729087415	1.3277689	3.561679608	0.00036849	0.00583616
BMSK0014318	22	30	28	0	3	0	13.4273467	4.703743037	1.17591655	4.000065335	6.33E-05	0.00123973
BMSK0009609	40	20	25	0	3	0	13.331444	4.698203583	1.16488798	4.033180588	5.50E-05	0.00111064
BMSK0000841	28	12	19	2	0	0	9.29138808	4.635735704	1.37396263	3.373989656	0.00074087	0.01030331
BMSK0009293	2035	1957	825	84	46	48	785.586355	4.571013758	0.33901504	13.48321808	1.96E-41	9.95E-39
BMSK0009842	56	29	14	0	4	0	15.2968337	4.489349156	1.0998652	4.081726706	4.47E-05	0.00092476
BMSK0009901	7165	6286	10064	19	1146	30	3923.42808	4.372050993	1.48412184	2.945884144	0.00322033	0.03404295
BMSK0009445	99	51	51	5	4	0	31.9129653	4.282753804	0.7261339	5.898022154	3.68E-09	1.76E-07
BMSK0015680	1470	165	570	37	20	40	327.240707	4.15815505	0.78579359	5.29166326	1.21E-07	4.44E-06
BMSK0009982	321	151	253	4	16	17	116.636857	4.130226027	0.4196758	9.841563586	7.45E-23	1.28E-20
BMSK0004276	972	1840	1671	143	34	54	783.766168	4.118519031	0.80045901	5.145196648	2.67E-07	9.23E-06
BMSK0012370	50	4	27	2	2	0	12.2086112	4.06942808	1.17231026	3.471289318	0.00051797	0.00768358
BMSK0000956	123	29	94	1	7	5	38.6323287	4.058541206	0.68441343	5.929955573	3.03E-09	1.46E-07
BMSK0012606	42	19	20	1	4	0	12.9039638	3.864713606	1.03832043	3.722081825	0.00019759	0.00336932
BMSK0014580	990	600	636	10	56	86	366.845746	3.725411555	0.7311966	5.09495195	3.49E-07	1.17E-05
BMSK0001508	29	26	16	0	2	3	11.9409268	3.700726215	1.04402343	3.544677344	0.00039309	0.00611979
BMSK0007302	34	23	2	1	1	2	9.53182679	3.663917131	1.24124893	2.951798827	0.00315929	0.03344743
BMSK0005027	11050	9428	8457	702	689	697	4903.78903	3.635619038	0.18697992	19.44390085	3.28E-84	4.28E-81
BMSK0012822	35816	54279	55708	6601	1524	2314	25762.6955	3.623312316	0.79840505	4.538188138	5.67E-06	0.00014584
BMSK0009169	47	14	46	0	4	4	17.4889954	3.585443696	0.90144621	3.97743501	6.97E-05	0.00135508
BMSK0013624	18	22	12	4	0	0	9.1018244	3.493377636	1.24554112	2.804706783	0.00503624	0.04761201
BMSK0014693	76	2	60	2	0	8	21.759939	3.475178054	1.03962229	3.342731396	0.00082958	0.01131281
BMSK0014704	29	26	16	2	4	0	12.0936547	3.453236337	1.01576342	3.399646274	0.00067473	0.00952874
BMSK0015448	208	54	56	16	6	2	49.7444175	3.403380461	0.64512974	5.275497729	1.32E-07	4.81E-06
BMSK0007680	1290	208	619	36	92	45	332.358638	3.391079422	0.63939214	5.303598884	1.14E-07	4.18E-06
BMSK0001189	266	249	240	8	38	22	130.620889	3.388980495	0.38748448	8.746106505	2.21E-18	2.70E-16
BMSK0001838	14	35	10	0	5	1	10.8090361	3.332250358	1.13911643	2.925293913	0.00344131	0.03575749
BMSK0015128	273	168	237	10	30	22	115.251478	3.317349331	0.37031162	8.958264196	3.30E-19	4.24E-17
BMSK0001312	766	56	276	34	48	12	168.388871	3.252806727	0.9407827	3.457553717	0.0005451	0.00793139
BMSK0011604	661	431	392	50	65	32	253.112626	3.172484941	0.28224433	11.2402078	2.59E-29	6.38E-27
BMSK0005604	44	23	43	6	2	3	18.9645731	3.114717522	0.78445459	3.970551718	7.17E-05	0.00138891
BMSK0005739	586	394	120	36	27	46	185.380225	3.110057382	0.42859096	7.256469894	3.97E-13	3.27E-11
BMSK0001333	3005	1568	1419	10	288	335	1007.99704	3.101895684	1.11094992	2.792111169	0.00523654	0.04919321
BMSK0014113	104	51	50	14	7	0	34.5795234	3.048052371	0.69837456	4.364495147	1.27E-05	0.0003035
BMSK0012099	768	485	489	66	56	70	301.30965	2.99090926	0.25820568	11.58343725	5.00E-31	1.38E-28
BMSK0010999	1771	1380	1345	232	193	96	792.941745	2.941362018	0.27804329	10.57879156	3.74E-26	7.58E-24
BMSK0001313	634	50	324	54	24	26	161.650853	2.928262324	0.90895557	3.221568174	0.00127491	0.0159329
BMSK0002557	47	46	30	6	5	4	22.0289399	2.886951042	0.69755215	4.138688451	3.49E-05	0.00074229
BMSK0012506	62	60	78	10	7	8	36.3341909	2.858809462	0.56218048	5.085216489	3.67E-07	1.22E-05
BMSK0002350	2663	961	2511	34	331	404	1059.93489	2.855591614	0.9097327	3.138934777	0.00169563	0.02001389
BMSK0012505	429	409	436	43	58	70	231.817543	2.769393338	0.28474341	9.72592597	2.34E-22	3.74E-20
BMSK0007723	29	26	20	8	2	0	13.6381182	2.697569617	0.96768284	2.78765884	0.00530904	0.0494606
BMSK0011641	4096	2362	2176	376	311	488	1520.21188	2.66274809	0.23902462	11.14005775	8.01E-29	1.92E-26
BMSK0016048	645	162	259	30	62	56	179.152549	2.610767275	0.37524491	6.957502173	3.46E-12	2.55E-10
BMSK0005619	122	26	60	16	8	4	35.155849	2.602188881	0.64813608	4.01488046	5.95E-05	0.00118471
BMSK0000960	162	31	97	11	19	11	49.2091506	2.598474122	0.54730405	4.747770698	2.06E-06	5.88E-05
BMSK0011640	1599	1107	1014	169	138	244	671.567034	2.561968924	0.25667516	9.981366888	1.84E-23	3.42E-21
BMSK0005260	49	50	27	6	12	2	23.2031448	2.557471482	0.70903659	3.606966865	0.0003098	0.00499343
BMSK0009845	1018	890	772	154	181	82	492.781124	2.548573114	0.26690209	9.548719211	1.31E-21	2.03E-19
BMSK0012117	96	53	27	5	17	6	30.9041473	2.495807558	0.63765358	3.914049293	9.08E-05	0.00170023
BMSK0003104	107	37	55	4	22	6	34.5783481	2.487113646	0.62078147	4.006423758	6.16E-05	0.00121466
BMSK0009891	356	193	289	12	101	30	151.417771	2.476613173	0.4515889	5.48422061	4.15E-08	1.66E-06
BMSK0009980	678	679	601	171	54	90	368.422819	2.437091893	0.3650448	6.676144748	2.45E-11	1.61E-09
BMSK0015264	279	367	312	82	20	55	183.76724	2.432435847	0.42913309	5.668255108	1.44E-08	6.24E-07

BMSK0013995	767	545	480	81	97	118	328.415584	2.428263928	0.24749586	9.811331605	1.01E-22	1.67E-20
BMSK0009839	2108	1566	1425	158	574	177	939.899957	2.409323602	0.31555359	7.635228107	2.25E-14	2.10E-12
BMSK0005110	58	54	60	14	16	0	32.4047875	2.405079683	0.69991239	3.437158305	0.00058785	0.00843235
BMSK0003290	18	40	32	4	2	10	17.8667973	2.391997396	0.82488008	2.899812286	0.00373386	0.03789102
BMSK0009306	1155	978	996	75	245	241	586.344145	2.375496529	0.30343835	7.828596838	4.93E-15	4.74E-13
BMSK0009942	243	216	202	7	74	43	124.247554	2.35382215	0.46223029	5.092314783	3.54E-07	1.18E-05
BMSK0003165	53	84	38	8	18	7	34.0028311	2.349611021	0.60455512	3.886512461	0.00010169	0.00187049
BMSK0006035	69	86	49	11	10	16	39.2192081	2.331686594	0.54247273	4.298255876	1.72E-05	0.0003986
BMSK0013470	102	38	28	1	25	6	29.4723044	2.258048086	0.7466473	3.024249995	0.0024925	0.02759601
BMSK0008915	21047	17035	17297	1819	7274	2159	10505.5403	2.243145682	0.57038875	3.932661132	8.40E-05	0.00158027
BMSK0009906	56	24	23	8	10	1	18.5261506	2.237963034	0.78108914	2.865182612	0.00416769	0.04137304
BMSK0014799	33	69	31	9	8	10	26.7980581	2.214772735	0.66039933	3.353687121	0.00079742	0.01097271
BMSK0012331	1676	939	1099	213	275	224	690.007178	2.205585364	0.19550341	11.28157004	1.62E-29	4.10E-27
BMSK0014768	855	742	448	88	98	209	388.209233	2.202428162	0.3317654	6.638510754	3.17E-11	2.05E-09
BMSK0009846	1020	598	629	179	214	50	419.304633	2.175133706	0.35068862	6.20246443	5.56E-10	3.05E-08
BMSK0002347	1079	329	714	143	137	122	385.677017	2.167891553	0.29596764	7.324758759	2.39E-13	2.06E-11
BMSK0013631	56	219	118	26	31	30	82.2744554	2.148858276	0.51465526	4.17533532	2.98E-05	0.00064786
BMSK0005638	70	68	72	26	15	2	41.0542588	2.127447916	0.62770625	3.389241271	0.00070086	0.00983688
BMSK0005148	86	72	30	19	20	0	35.7709026	2.114643578	0.73378254	2.881839578	0.00395361	0.03974957
BMSK0002375	317	243	264	94	35	36	158.793067	2.108397305	0.39518667	5.335193393	9.54E-08	3.57E-06
BMSK0005445	210	110	184	32	56	18	95.2689027	2.108129756	0.39476848	5.34016731	9.29E-08	3.49E-06
BMSK0004257	51	39	23	2	17	6	21.4379781	2.08800091	0.73238303	2.850968444	0.00435863	0.04294917
BMSK0000074	378	155	244	46	49	65	144.751975	2.066995907	0.32636433	6.333400244	2.40E-10	1.38E-08
BMSK0004278	48	33	29	9	2	12	21.1608771	2.032566932	0.72506596	2.803285556	0.00505849	0.04772344
BMSK0012731	168	144	286	38	54	44	118.925274	2.026014051	0.37134939	5.455816314	4.87E-08	1.94E-06
BMSK0000897	75	32	18	7	8	11	22.8364584	2.02180526	0.68368911	2.957199731	0.00310447	0.03300941
BMSK0016032	95	31	53	8	13	17	33.0424181	2.018339805	0.56838652	3.550998698	0.00038377	0.00603647
BMSK0002516	5452	4145	4212	706	1147	1262	2688.432	2.005236775	0.20470268	9.795849975	1.17E-22	1.91E-20
BMSK0001203	150	106	74	18	22	33	63.4419073	1.997790845	0.4312631	4.63241777	3.61E-06	9.73E-05
BMSK0001456	3213	1799	2382	242	1038	453	1416.91585	1.994424322	0.30823713	6.470421997	9.77E-11	5.94E-09
BMSK0001980	304	100	154	24	54	44	102.946734	1.991069029	0.38510655	5.170176966	2.34E-07	8.17E-06
BMSK0009430	42	106	43	24	14	8	40.230816	1.964993437	0.62368528	3.150616984	0.00162926	0.01939592
BMSK0011967	823	383	398	67	236	74	301.986964	1.955366226	0.35125052	5.56687068	2.59E-08	1.07E-06
BMSK0001426	67	38	52	14	6	15	30.4302631	1.949654409	0.58797206	3.315896327	0.0009135	0.01221548
BMSK0008784	78	54	46	9	31	4	34.5437415	1.924407834	0.61794097	3.114226022	0.00184428	0.02148835
BMSK0015156	107	97	84	26	6	35	57.6089476	1.900824305	0.53861105	3.529122338	0.00041694	0.00640806
BMSK0002496	85	35	21	6	17	10	26.1161307	1.89563968	0.65211019	2.906931541	0.00364993	0.0372926
BMSK0014561	398	201	254	62	62	77	164.637903	1.883978737	0.29347164	6.419627883	1.37E-10	7.94E-09
BMSK0011156	217	126	208	52	43	37	108.489128	1.881896185	0.3477554	5.411551261	6.25E-08	2.40E-06
BMSK0015552	121	30	95	20	24	15	46.7876744	1.848697654	0.52005892	3.554785001	0.00037829	0.00596049
BMSK0013542	114	76	52	24	14	20	47.3638868	1.848238087	0.48750244	3.791238662	0.0001499	0.00262479
BMSK0012605	1106	708	830	185	286	192	521.459388	1.845361581	0.20503744	9.00012009	2.25E-19	2.94E-17
BMSK0004472	51	52	54	16	14	10	32.0387473	1.837354333	0.54875124	3.348246327	0.00081325	0.01115677
BMSK0005383	48	44	54	12	12	14	29.8240271	1.802649542	0.55699554	3.236380572	0.00121056	0.01527514
BMSK0013274	404	1080	785	236	143	234	490.801955	1.800168513	0.62501259	2.880211618	0.00397408	0.03988511
BMSK0000952	2668	1101	1909	407	556	484	1106.38614	1.77975558	0.22060213	8.067717084	7.16E-16	7.34E-14
BMSK0011318	80	85	77	22	19	23	49.8593213	1.779474566	0.4540003	3.919544899	8.87E-05	0.00166535
BMSK0008905	107	76	94	16	32	26	55.7385317	1.770973336	0.42245081	4.19214097	2.76E-05	0.00060747
BMSK0014385	756	297	315	70	114	160	261.567999	1.767469921	0.3252452	5.434269115	5.50E-08	2.13E-06
BMSK0001126	16158	10167	14436	2078	4136	4685	8181.62905	1.75934146	0.23746439	7.408864449	1.27E-13	1.11E-11
BMSK0009199	85	49	62	25	18	8	39.0792256	1.747407644	0.53367259	3.274306551	0.00105922	0.01378492
BMSK0014435	129	67	82	20	17	35	54.9673651	1.739862586	0.45587759	3.816512638	0.00013535	0.00241174
BMSK0005710	270	77	90	14	53	47	82.1543531	1.720587065	0.47453012	3.625875352	0.00028798	0.00467488
BMSK0015668	58	34	74	16	20	10	33.847669	1.710258217	0.55277431	3.093953861	0.00197508	0.02263652
BMSK0014513	263	125	191	46	70	42	114.972709	1.702578363	0.32791374	5.192153129	2.08E-07	7.35E-06
BMSK0000912	162	121	95	62	28	10	76.6083269	1.701692349	0.51695194	3.291780595	0.00099555	0.01312489
BMSK0015217	1337	548	673	77	317	313	499.734838	1.682230468	0.35359561	4.757498235	1.96E-06	5.66E-05
BMSK0015906	2795	608	1206	355	356	471	871.031499	1.681810432	0.31874955	5.27627544	1.32E-07	4.81E-06
BMSK0011000	635	633	582	188	194	145	385.525717	1.678122241	0.24478239	6.855567555	7.10E-12	4.95E-10
BMSK0009578	20453	23120	23242	4759	8500	6490	14085.0278	1.670464198	0.21574082	7.742921344	9.72E-15	9.23E-13
BMSK0012604	88	58	62	15	38	8	42.1534082	1.659809926	0.53306347	3.113719123	0.00184745	0.02149785
BMSK0013141	99	60	107	9	52	19	54.3393313	1.658114129	0.51991066	3.189228971	0.00142653	0.01753938
BMSK0001681	2339	3560	2924	823	817	1001	1901.95205	1.63708805	0.26264671	6.233042308	4.57E-10	2.56E-08
BMSK0013884	620	521	456	99	168	200	330.434205	1.636072731	0.2657714	6.155939719	7.46E-10	3.94E-08
BMSK0013456	681	382	753	155	222	159	373.92501	1.613666511	0.26014193	6.203023521	5.54E-10	3.05E-08
BMSK0002346	75	44	88	33	16	10	42.8901508	1.612369119	0.55449311	2.907825365	0.00363952	0.0372926
BMSK0014578	503	176	457	97	128	103	228.589087	1.604756763	0.31516172	5.091851827	3.55E-07	1.18E-05
BMSK0013403	425	84	206	59	84	56	137.335534	1.598216747	0.39658905	4.029906342	5.58E-05	0.00111947
BMSK0001271	94	211	202	40	84	41	112.283759	1.597957218	0.43089887	3.708427511	0.00020855	0.00353644
BMSK0015167	396	190	281	64	87	104	175.862167	1.577556167	0.29379264	5.369624448	7.89E-08	2.97E-06
BMSK0011272	285	142	182	34	68	80	123.515618	1.565982632	0.34407639	4.551264356	5.33E-06	0.0001382
BMSK0004081	245	223	190	61	37	99	138.833158	1.5619831	0.37793199	4.132974078	3.58E-05	0.00075799
BMSK0014205	295	142	136	54	44	68	115.217595	1.558028857	0.35370749	4.404851195	1.06E-05	0.00025684
BMSK0012607	778	326	330	188	130	92	285.459382	1.553930396	0.32651	4.75921224	1.94E-06	5.63E-05
BMSK0011959	376	300	264	99	100	88	196.714079	1.551048757	0.26565474	5.838588608	5.26E-09	2.46E-07
BMSK0014315	733	614	1137	223	312	251	530.865231	1.546375262	0.26808916	5.768137874	8.02E-09	3.58E-07
BMSK0011031	1139	1088	956	452	168	330	676.056094	1.545708101	0.32783061	4.714959601	2.42E-06	6.79E-05
BMSK0014387	402	374	297	111	113	110	227.380604	1.535824097	0.26484089	5.799044443	6.67E-09	3.04E-07
BMSK0010380	829	764	1058	230	381	242	567.367132	1.534630505	0.24385062	6.293322255	3.11E-10	1.77E-08
BMSK0007152	118	60	118	33	21	35	61.4789924	1.533033165	0.44362308	3.45571103	0.00054884	0.00796041
BMSK0002376	4672	2807	4546	1083	1367	1274	2509.00733	1.531597568	0.19377998	7.903796886	2.71E-15	2.65E-13
BMSK0011424	2011	1235	1760	378	591	588	1042.1602	1.53132913	0.20641283	7.418769061	1.18E-13	1.04E-11
BMSK0000910	2033	2243	2717	1088	671	432	1514.08988	1.524157456	0.51925392	2.935283483	0.00333243	0.03488561
BMSK0014384	109	92	136	22	33	52	71.9659353	1.522860386	0.42410376	3.590773139	0.0003297	0.00526767
BMSK0008909	147	82	91	40	55	5	65.7159893	1.522320629	0.54579625	2.789173857	0.00528427	0.04945323
BMSK0015701	9019	5565	5132	1341	2508	2312	4066.7999	1.518296831	0.20768608	7.310537302	2.66E-13	2.27E-11
BMSK0001125	701	484	556	154	181	208	364.646059	1.516516195	0.22561119	6.721812733	1.79E-11	1.20E-09
BMSK0012302	126	56	62	26	34	16	49.5227394	1.486379694	0.46195568	3.217580743	0.00129277	0.01608991
BMSK0007073	1009	471	502	213	253	152	403.988924	1.478493786	0.2557255	5.781565759	7.40E-09	3.34E-07
BMSK0015700	20775	16028	12007	5245	5109	5198	10289.2305	1.474901032	0.19724583	7.47747648	7.58E-14	6.78E-12

BMSK0015064	1443	762	1542	251	650	348	788.336952	1.470877078	0.27926947	5.266873905	1.39E-07	5.00E-06
BMSK0003029	238	128	123	38	78	43	100.809134	1.459657775	0.34973497	4.173611129	3.00E-05	0.00064968
BMSK0014715	1784	832	1515	432	544	355	860.132135	1.458098827	0.23092032	6.314294027	2.71E-10	1.56E-08
BMSK0014705	635	139	392	117	151	98	233.832317	1.439681013	0.35404274	4.066404587	4.77E-05	0.00098323
BMSK0001007	94	98	103	45	35	19	64.5430834	1.435741087	0.44150026	3.251959754	0.00114612	0.0146649
BMSK0014673	108	50	75	21	38	18	48.3253956	1.434710726	0.45707341	3.138906534	0.0016958	0.02001389
BMSK0004631	110	131	136	27	64	42	83.1087994	1.428071552	0.39279228	3.635691507	0.00027724	0.00451647
BMSK0005073	286	213	180	61	125	48	144.474231	1.416268969	0.3329595	4.253577249	2.10E-05	0.00047391
BMSK0005300	396	178	239	75	115	80	168.647679	1.405544884	0.28689501	4.899161187	9.62E-07	2.96E-05
BMSK0015437	687	341	335	158	134	149	282.635182	1.405016778	0.26982856	5.207072146	1.92E-07	6.81E-06
BMSK0013018	88	92	132	45	38	25	69.074519	1.402597468	0.43010215	3.261079845	0.00110989	0.01428139
BMSK0008342	899	392	454	255	184	128	361.194746	1.382092651	0.30236575	4.570929863	4.86E-06	0.00012729
BMSK0001392	239	136	117	16	124	38	103.093291	1.377154045	0.48204341	2.85690874	0.00427789	0.04228296
BMSK0013380	83	57	49	13	30	23	40.2637711	1.376026466	0.48466856	2.839108152	0.00452398	0.04442658
BMSK0007275	216	83	120	43	71	28	86.498077	1.375100336	0.39766769	3.457913167	0.00054438	0.00793139
BMSK0003781	854	599	750	224	296	246	475.236036	1.374859443	0.20335203	6.760982286	1.37E-11	9.26E-10
BMSK0014463	517	366	334	150	130	135	261.144649	1.369745176	0.25331582	5.407262716	6.40E-08	2.44E-06
BMSK0014062	363	265	218	86	111	102	182.536144	1.338949322	0.2681451	4.993376029	5.93E-07	1.89E-05
BMSK0003597	107	61	140	26	28	55	67.2420238	1.332756668	0.45903029	2.903417716	0.00369114	0.03762488
BMSK0000710	243	51	196	32	84	56	101.53476	1.329487483	0.44885353	2.961962846	0.00305685	0.03257899
BMSK0010591	2803	1528	1115	542	1037	367	1146.29635	1.324171981	0.29053494	4.55770303	5.17E-06	0.00013469
BMSK0003260	196	82	90	46	54	28	76.8226545	1.312684781	0.39957322	3.28521713	0.00101904	0.01335731
BMSK0014696	317	683	351	242	141	126	315.455358	1.302756727	0.38671505	3.368776926	0.00075503	0.01045234
BMSK0000445	227	158	212	83	70	66	131.61394	1.27621484	0.30987517	4.118480462	3.81E-05	0.00080354
BMSK0014464	691	709	522	255	241	223	430.60141	1.275293385	0.24736223	5.155570413	2.53E-07	8.80E-06
BMSK0009406	198	129	87	36	71	47	89.2320775	1.270855721	0.37029889	3.431972851	0.00059921	0.00854152
BMSK0013435	2706	2062	2036	807	937	796	1500.82127	1.266073698	0.17363109	7.291745471	3.06E-13	2.56E-11
BMSK0013078	2406	1963	2817	798	954	973	1608.53875	1.262016198	0.20838565	6.056156812	1.39E-09	6.95E-08
BMSK0007208	164	108	150	27	91	46	92.7289268	1.26186818	0.38915325	3.242599656	0.00118445	0.01501704
BMSK0015957	107	65	72	34	39	18	53.1545614	1.254039402	0.43859031	2.859250132	0.00424644	0.04206325
BMSK0014681	638	522	600	182	284	213	392.947393	1.247780875	0.22019657	5.666667977	1.46E-08	6.27E-07
BMSK0010540	249	160	255	104	62	79	147.183508	1.24618807	0.33792639	3.687750107	0.00022625	0.00380118
BMSK0015416	226864	280586	421709	133133	86412	137811	214500.43	1.244037469	0.29475554	4.220573592	2.44E-05	0.00053828
BMSK0011693	2232	2250	2260	768	717	1092	1526.26786	1.243339296	0.23971643	5.186708782	2.14E-07	7.54E-06
BMSK0015771	2979	2975	4412	1392	1401	1226	2362.69811	1.242962097	0.23858045	5.209823689	1.89E-07	6.74E-06
BMSK0007523	234	97	80	48	61	40	86.2540071	1.241802745	0.3919396	3.168352366	0.00153306	0.01847565
BMSK0009508	1610	980	1013	392	595	378	785.416446	1.240393087	0.19871086	6.242200688	4.31E-10	2.43E-08
BMSK0009145	474	189	278	101	154	94	200.403821	1.239111724	0.2891781	4.284943174	1.83E-05	0.00042061
BMSK0000481	4061	2991	4077	1295	1664	1317	2481.50957	1.238809448	0.18164394	6.819987931	9.10E-12	6.25E-10
BMSK0010736	384	167	236	97	141	57	168.687441	1.235052549	0.32705742	3.776255986	0.0001592	0.00277178
BMSK0006032	2291	1263	1481	455	860	604	1093.756	1.234817825	0.20352801	6.067065726	1.30E-09	6.57E-08
BMSK0000078	206	103	115	53	51	53	91.5388476	1.226491854	0.34585075	3.546303893	0.00039068	0.00610846
BMSK0014316	68	88	120	31	49	32	63.9882349	1.221144242	0.43557073	2.803549805	0.00505434	0.04772344
BMSK0003840	625	282	283	155	155	133	254.972812	1.203408749	0.27542504	4.369278653	1.25E-05	0.00029771
BMSK0001679	187	284	275	83	142	88	175.134038	1.188031779	0.3334415	3.562939153	0.00036673	0.0058185
BMSK0008722	596	360	305	140	206	148	276.810282	1.180138958	0.24810007	4.756705376	1.97E-06	5.66E-05
BMSK0008378	7262	2136	2631	1823	1419	1171	2522.05451	1.16302844	0.29387585	3.957550243	7.57E-05	0.00144522
BMSK0008922	128	68	156	39	56	47	79.1424865	1.161625079	0.39239135	2.960373782	0.00307266	0.03270931
BMSK0008395	397	318	338	108	178	148	239.264628	1.147554569	0.24632155	4.658766444	3.18E-06	8.72E-05
BMSK0015478	344	308	254	146	98	121	207.31667	1.137149845	0.29785326	3.817819068	0.00013464	0.0024037
BMSK0001032	212	141	151	74	57	71	113.564124	1.129416169	0.32397473	3.48612426	0.00049007	0.00738998
BMSK0003439	150	194	163	103	46	59	119.467096	1.126771419	0.40378609	2.790515699	0.00526241	0.04934122
BMSK0011094	1233	786	705	463	434	203	609.251734	1.121840619	0.2827158	3.968086034	7.25E-05	0.00139827
BMSK0013799	272	158	84	46	112	54	112.679753	1.119947071	0.39622327	2.826555511	0.00470516	0.04545732
BMSK0003596	274	176	186	83	88	88	143.089628	1.118918394	0.2821188	3.966124897	7.31E-05	0.00140599
BMSK0014118	405	240	261	90	161	125	202.782409	1.111059363	0.26077465	4.260610999	2.04E-05	0.00046151
BMSK0005860	417	269	344	164	126	130	233.673263	1.106998954	0.26952446	4.107229993	4.00E-05	0.00084174
BMSK0006071	1730	1270	1749	604	548	814	1089.89403	1.104514635	0.23415555	4.717012335	2.39E-06	6.76E-05
BMSK0010150	454	248	344	161	155	112	234.957402	1.102476194	0.26640706	4.138314408	3.50E-05	0.00074229
BMSK0001817	196	101	84	48	62	45	83.9563239	1.10098602	0.36597646	3.008351984	0.00262669	0.02859082
BMSK0013077	984	1056	1072	368	591	399	729.51206	1.098763913	0.22653226	4.850364056	1.23E-06	3.74E-05
BMSK0014567	919	824	440	270	252	383	499.830143	1.098411667	0.28905712	3.799981395	0.00014471	0.0025535
BMSK0009148	2512	1792	2302	748	1152	917	1513.73563	1.092291024	0.18174663	6.009965784	1.86E-09	9.15E-08
BMSK0012982	492	461	482	175	198	239	334.06843	1.091021549	0.24721336	4.413279162	1.02E-05	0.00024836
BMSK0002226	172	103	138	41	91	47	93.711769	1.075301015	0.35336596	3.043023757	0.00234214	0.02621196
BMSK0012351	320	173	264	120	89	104	171.621928	1.073337614	0.30053868	3.571379273	0.00035511	0.00564396
BMSK0014459	24102	17463	25148	5699	15175	8839	15422.3849	1.071851056	0.24116122	4.444541597	8.81E-06	0.0002177
BMSK0014546	357	152	198	68	128	100	156.264501	1.071194239	0.30493051	3.512912591	0.00044322	0.00678444
BMSK0007101	2336	1706	2034	809	969	828	1399.49562	1.069166073	0.17433871	6.1326945	8.64E-10	4.45E-08
BMSK0010037	1711	1278	1021	726	510	443	918.564618	1.061513955	0.25430032	4.174253296	2.99E-05	0.00064939
BMSK0006043	6195	4923	8292	3387	2173	2725	4549.1168	1.058002793	0.26389919	4.009117209	6.09E-05	0.00120872
BMSK0000846	1951	1142	1171	559	608	630	964.202019	1.056245416	0.19385868	5.448532987	5.08E-08	2.01E-06
BMSK0003546	905	349	504	157	270	308	388.386622	1.053505293	0.27999081	3.762642368	0.00016813	0.00289516
BMSK0015674	294	222	181	119	61	111	160.352911	1.052724569	0.34496941	3.051646163	0.0022759	0.02560179
BMSK0005702	891	932	1245	231	531	627	729.132205	1.052103588	0.30283411	3.474191193	0.0005124	0.00765071
BMSK0010319	248	168	149	83	103	58	129.026405	1.048740832	0.31126663	3.369268433	0.00075368	0.01044958
BMSK0014280	619	317	331	175	162	193	284.440258	1.044126002	0.25828119	4.042594042	5.29E-05	0.00107171
BMSK0006832	1023	915	1013	492	315	474	694.859069	1.032120161	0.26142534	3.948049448	7.88E-05	0.0014944
BMSK0001289	3668	3809	2277	1378	1437	1511	2298.34871	1.030106414	0.23135189	4.452552477	8.49E-06	0.00021097
BMSK0010281	1145	890	1216	338	706	436	761.174731	1.030045621	0.23273253	4.425877279	9.61E-06	0.00023493
BMSK0001502	1807	1866	1798	706	955	826	1300.6718	1.021399254	0.20228124	5.049401775	4.43E-07	1.43E-05
BMSK0010992	723	744	584	326	314	278	486.127316	1.019481251	0.23922891	4.261530345	2.03E-05	0.00046076
BMSK0013902	440	443	573	172	294	206	347.379909	1.019289189	0.26110337	3.903776436	9.47E-05	0.00175963
BMSK0009085	311	176	264	114	116	98	172.843854	1.017540549	0.27787129	3.661913226	0.00025034	0.00411882
BMSK0014379	229	131	106	63	68	70	105.61703	1.014299703	0.33395356	3.037247728	0.00238749	0.0265623
BMSK0008912	1793	892	1432	473	779	583	942.481923	1.003244625	0.2106831	4.761865584	1.92E-06	5.57E-05
BMSK0015170	225	123	117	352	306	149	211.79531	-1.001606825	0.33352182	-3.00312231	0.00267225	0.02902254
BMSK0008336	618	536	575	994	1276	898	818.950158	-1.005018829	0.19879989	-5.05542948	4.29E-07	1.39E-05
BMSK0015169	1024	429	758	1280	1271	1299	1007.74223	-1.008960354	0.22772886	-4.43053351	9.40E-06	0.00023115
BMSK0006033	214	254	244	420	499	412	345.215725	-1.014737679	0.25314994	-4.00844535	6.11E-05	0.00120954

BMSK0005741	1244	1175	1515	1823	3354	2266	1891.19086	-1.01519197	0.22079278	-4.59794007	4.27E-06	0.00011382
BMSK0004181	810	383	252	511	1312	774	649.305907	-1.021665252	0.30557039	-3.34346946	0.00082738	0.01129966
BMSK0012179	324	292	214	465	476	572	394.985424	-1.024282725	0.24567341	-4.16928603	3.06E-05	0.00066056
BMSK0012173	1721	1077	1167	2139	2848	2216	1851.4675	-1.026633959	0.16663338	-6.16103413	7.23E-10	3.83E-08
BMSK0000520	647	174	514	467	947	970	606.200439	-1.029544752	0.32364725	-3.18107058	0.00146732	0.01784847
BMSK0007177	678	451	580	928	1279	963	810.816654	-1.041094958	0.186329	-5.58740151	2.30E-08	9.56E-07
BMSK0012680	94988	66836	65161	65110	158048	200281	107431.359	-1.041369752	0.27363173	-3.8057346	0.00014138	0.0024997
BMSK0004809	68	54	62	114	96	127	88.2904489	-1.042455263	0.35499592	-2.93652745	0.00331909	0.03484476
BMSK0015226	691	454	429	979	1030	859	741.714934	-1.044565467	0.19937065	-5.23931408	1.61E-07	5.77E-06
BMSK0000887	58	51	77	92	163	103	90.4308918	-1.048911523	0.36169026	-2.90002701	0.00373131	0.03789102
BMSK0015800	1438	1267	1410	2193	2864	2717	1994.62877	-1.049795333	0.18537022	-5.66323617	1.49E-08	6.33E-07
BMSK0009848	1302	884	922	1578	2366	1867	1477.96535	-1.054197928	0.17131171	-6.15368268	7.57E-10	3.97E-08
BMSK0013199	82	56	60	144	98	118	94.6264604	-1.058534803	0.35705158	-2.96465516	0.00303022	0.03234118
BMSK0008938	182234	75457	83140	194599	205006	207676	156622.507	-1.065277667	0.21561762	-4.94058735	7.79E-07	2.43E-05
BMSK0008155	254	179	191	396	404	366	300.336712	-1.071587977	0.22881361	-4.68323521	2.82E-06	7.83E-05
BMSK0011325	877	343	419	775	1527	734	754.957926	-1.0719928	0.26605273	-4.02924938	5.60E-05	0.00111947
BMSK0002040	2057	1308	1167	2811	2836	2713	2154.77342	-1.072841916	0.19004055	-5.64533151	1.65E-08	7.00E-07
BMSK0006627	213	94	73	110	258	326	174.794658	-1.073092878	0.3783926	-2.8359246	0.00456932	0.04477544
BMSK0014775	480	264	206	606	562	556	445.568778	-1.074433755	0.25269425	-4.25191215	2.12E-05	0.00047618
BMSK0001550	1125	736	803	1871	2319	865	1279.20811	-1.078604279	0.26941258	-4.0035409	6.24E-05	0.00122691
BMSK0007228	629	385	485	674	1205	983	719.429104	-1.080624391	0.20599367	-5.24591055	1.56E-07	5.59E-06
BMSK0013590	582	279	324	554	871	779	556.959085	-1.081259469	0.22257808	-4.85788829	1.19E-06	3.61E-05
BMSK0013320	274	301	126	338	532	499	346.101644	-1.087186843	0.30778809	-3.53225769	0.00041203	0.00636028
BMSK0011052	261	159	150	380	372	313	273.227324	-1.092282669	0.24820223	-4.40077699	1.08E-05	0.00025984
BMSK0015277	720	427	419	764	1350	897	751.378091	-1.099474976	0.20873175	-5.26740638	1.38E-07	5.00E-06
BMSK0001493	154	46	92	189	224	127	136.432762	-1.103568226	0.35265455	-3.12931802	0.00175213	0.02054581
BMSK0008633	153	148	134	345	155	329	218.540595	-1.11913505	0.35249463	-3.17489953	0.00149888	0.01815978
BMSK0005067	2685	2702	2699	4261	7179	5006	4092.16539	-1.125342722	0.1986177	-5.66587327	1.46E-08	6.27E-07
BMSK0001435	102	72	56	142	154	149	112.958859	-1.128205202	0.31520456	-3.57927943	0.00034454	0.00548563
BMSK0003863	3906	4623	4788	9355	9631	7909	6832.98357	-1.129453039	0.22242148	-5.07798538	3.81E-07	1.25E-05
BMSK0004526	391	241	169	470	605	470	388.42324	-1.130555182	0.24685229	-4.57988539	4.65E-06	0.00012267
BMSK0002189	1166	897	945	1155	2636	2278	1498.61495	-1.132095381	0.230615	-4.90902757	9.15E-07	2.82E-05
BMSK0001494	214	155	136	240	405	359	250.078624	-1.138599417	0.25116233	-4.53332082	5.81E-06	0.00014838
BMSK0008477	1311	498	879	1003	2710	1648	1300.53199	-1.153795988	0.27235637	-4.2363467	2.27E-05	0.00050552
BMSK0015166	1255	978	1202	1930	2859	2211	1740.69311	-1.158109601	0.18040222	-6.41959725	1.37E-10	7.94E-09
BMSK0004680	244	160	183	389	552	247	293.701405	-1.16264628	0.27235274	-4.26889879	1.96E-05	0.00044691
BMSK0003810	2566	1970	1805	2823	5010	5088	3201.13486	-1.166244593	0.20775297	-5.61361225	1.98E-08	8.29E-07
BMSK0010294	237	170	262	387	468	501	340.483264	-1.167985951	0.24519484	-4.76350127	1.90E-06	5.55E-05
BMSK0008399	625	539	445	862	1309	1156	823.962107	-1.18251269	0.20284238	-5.82971226	5.55E-09	2.57E-07
BMSK0005309	633	222	654	846	1134	1042	751.791947	-1.182984919	0.28380457	-4.1683082	3.07E-05	0.00066184
BMSK0002729	404	300	244	602	772	566	481.531487	-1.190031698	0.21436805	-5.55134826	2.83E-08	1.16E-06
BMSK0012277	2054	1176	1402	2716	3407	3253	2333.20364	-1.19590108	0.1712819	-6.98206342	2.91E-12	2.18E-10
BMSK0003486	3380	1866	2210	4800	5715	4546	3746.63319	-1.197549101	0.17127484	-6.99197327	2.71E-12	2.04E-10
BMSK0004014	178	140	125	273	445	214	227.41407	-1.200503772	0.27793615	-4.31935095	1.56E-05	0.00036513
BMSK0001869	5378	4202	3606	4664	12535	10780	6773.54687	-1.202334555	0.2502313	-4.80489279	1.55E-06	4.65E-05
BMSK0014443	70	54	33	111	118	92	80.1106429	-1.203233946	0.37377161	-3.21916894	0.00128563	0.01602292
BMSK0013797	55	34	28	70	87	81	59.0780606	-1.204108877	0.39971887	-3.01238939	0.002592	0.02838752
BMSK0005620	497	114	160	505	562	403	365.146799	-1.207731484	0.33616236	-3.59270288	0.00032727	0.00523797
BMSK0002017	5717	6097	5408	14644	12340	9160	9099.27598	-1.210282538	0.23501091	-5.14989929	2.61E-07	9.04E-06
BMSK0002823	72	44	42	84	96	142	80.416184	-1.215306477	0.37812964	-3.21399419	0.00130902	0.01627005
BMSK0001554	173849	113480	81926	262170	257099	229929	187382.949	-1.215501607	0.20888136	-5.81910054	5.92E-09	2.73E-07
BMSK0014763	290	182	190	368	607	412	338.393782	-1.218820196	0.22573765	-5.39927747	6.69E-08	2.54E-06
BMSK0009242	230	91	118	135	487	297	217.89436	-1.221602849	0.3520575	-3.4698958	0.00052066	0.00769852
BMSK0005074	191	161	222	321	564	358	302.198196	-1.223204787	0.25739348	-4.75227564	2.01E-06	5.77E-05
BMSK0002664	107	112	114	196	356	182	177.408368	-1.232299925	0.30357748	-4.05926002	4.92E-05	0.00100248
BMSK0015260	313	168	277	532	570	485	392.891127	-1.243460256	0.23380419	-5.31838315	1.05E-07	3.87E-06
BMSK0001552	2794	1696	1643	4168	5527	3324	3176.12758	-1.255181062	0.19417079	-6.46431448	1.02E-10	6.12E-09
BMSK0007720	126	128	35	226	184	202	153.181878	-1.255875987	0.40205184	-3.12366679	0.00178613	0.0208908
BMSK0001159	164	126	146	184	462	320	230.750048	-1.255968143	0.28825204	-4.35718733	1.32E-05	0.000313
BMSK0006532	1441	496	782	1652	2472	1518	1368.10704	-1.259439398	0.24295228	-5.18389615	2.17E-07	7.62E-06
BMSK0015759	75	38	28	79	123	96	72.1562692	-1.269159595	0.39174265	-3.23977895	0.00119622	0.01511517
BMSK0004707	491	395	482	786	1107	1115	734.400293	-1.273345724	0.20736341	-6.14064795	8.22E-10	4.26E-08
BMSK0001742	96	46	20	118	102	110	81.9066631	-1.273741085	0.43932917	-2.89928637	0.00374013	0.03791246
BMSK0013202	77	138	46	261	217	110	146.034673	-1.276319672	0.4375113	-2.91722674	0.00353159	0.03642785
BMSK0016035	360	141	201	585	433	426	360.31662	-1.282086338	0.28264033	-4.53610552	5.73E-06	0.00014685
BMSK0009297	3139	2198	1657	4195	6509	4605	3696.15239	-1.28455934	0.19573968	-6.5625903	5.29E-11	3.33E-09
BMSK0014323	425	355	387	635	1063	923	632.306802	-1.292402843	0.21111903	-6.12167853	9.26E-10	4.75E-08
BMSK0003961	114	43	44	155	168	100	102.954099	-1.300828015	0.37471827	-3.4714828	0.00051759	0.00768358
BMSK0000579	848	667	428	1538	1392	1308	1044.45389	-1.305561375	0.23544463	-5.5450888	2.94E-08	1.20E-06
BMSK0011643	328	190	159	548	443	456	357.947318	-1.309655495	0.26171171	-5.00419148	5.61E-07	1.80E-05
BMSK0005850	24	29	38	78	62	71	51.9843337	-1.348210407	0.46050363	-2.92768683	0.00341494	0.03560512
BMSK0001219	290	102	116	349	489	278	265.826696	-1.353605251	0.30138903	-4.49122262	7.08E-06	0.00017847
BMSK0013883	72	55	64	160	170	110	106.338117	-1.357334827	0.33143471	-4.09533096	4.22E-05	0.00088008
BMSK0011449	84	45	76	176	207	92	113.449392	-1.374852965	0.36346885	-3.78258815	0.00015521	0.00270735
BMSK0014631	1930	836	1173	2249	3048	3806	2170.67285	-1.413481518	0.22912314	-6.16909103	6.87E-10	3.66E-08
BMSK0007001	340	228	176	605	486	637	419.33745	-1.418138501	0.25844369	-5.48722424	4.08E-08	1.64E-06
BMSK0005880	242	165	129	147	760	445	304.642315	-1.434617522	0.37137515	-3.86298741	0.00011201	0.00203557
BMSK0000777	200	77	100	246	333	303	208.26155	-1.435898192	0.28209819	-5.09006531	3.58E-07	1.19E-05
BMSK0006453	727	465	445	1460	1262	1171	935.650217	-1.444350811	0.21405484	-6.7475736	1.50E-11	1.01E-09
BMSK0001501	244	180	170	468	450	519	344.143647	-1.449555965	0.2347751	-6.17423221	6.65E-10	3.57E-08
BMSK0008878	1279	980	1112	2708	2809	2763	1972.5617	-1.455589724	0.1799381	-8.08939151	6.00E-16	6.29E-14
BMSK0005068	1526	847	1109	2205	4094	2371	2002.22019	-1.462765418	0.20316593	-7.19985574	6.03E-13	4.82E-11
BMSK0015682	178	135	108	256	426	386	248.306927	-1.48918335	0.25806242	-5.77063236	7.90E-09	3.55E-07
BMSK0013795	171	33	91	256	256	185	164.283944	-1.497979601	0.37956221	-3.94659837	7.93E-05	0.00150036
BMSK0015595	563	253	355	889	990	992	676.493785	-1.498148277	0.21661717	-6.9161105	4.64E-12	3.31E-10
BMSK0014927	41	38	20	106	70	72	59.6031549	-1.510150846	0.45277753	-3.33530427	0.00085206	0.01151608
BMSK0002840	52	34	51	114	116	121	82.6765434	-1.524265819	0.35700777	-4.26955923	1.96E-05	0.00044671
BMSK0012987	1820	1075	979	4191	4120	1461	2288.29879	-1.525052962	0.30169947	-5.0548745	4.31E-07	1.39E-05
BMSK0011173	59	98	36	142	204	178	121.707509	-1.528994533	0.3917201	-3.90328328	9.49E-05	0.00175964
BMSK0013303	103	39	50	162	136	174	111.690139	-1.536068983	0.35414209	-4.3374369	1.44E-05	0.000339

BMSK0008951	47	24	9	70	68	60	46.4377616	-1.53847147	0.51624773	-2.98010315	0.00288151	0.03103665
BMSK0001826	864	451	555	1882	1417	1389	1113.45209	-1.542800297	0.23186747	-6.65380233	2.86E-11	1.86E-09
BMSK0009915	126	92	28	298	156	160	147.726079	-1.55640778	0.43841683	-3.55006398	0.00038514	0.00604752
BMSK0001888	280	162	242	616	633	548	418.940927	-1.570059983	0.22654358	-6.93049865	4.19E-12	3.06E-10
BMSK0011176	72	46	39	121	176	127	96.8067871	-1.596629787	0.33301569	-4.7944581	1.63E-06	4.83E-05
BMSK0008358	84	155	94	229	453	292	219.80393	-1.598129802	0.34120928	-4.68372316	2.82E-06	7.83E-05
BMSK0003984	244	96	118	306	624	304	275.772018	-1.606828475	0.29793775	-5.39316841	6.92E-08	2.62E-06
BMSK0004407	25	11	14	34	54	47	30.6621538	-1.615747444	0.53898229	-2.99777467	0.00271959	0.02939667
BMSK0011175	24	16	12	39	41	60	32.4851102	-1.61736361	0.53992545	-2.99553137	0.00273967	0.02957872
BMSK0016013	2011	2717	2961	8864	5852	6866	5089.3294	-1.620787481	0.2755089	-5.88288602	4.03E-09	1.92E-07
BMSK0003816	41	36	18	82	100	82	60.4913582	-1.630707398	0.42255491	-3.85916088	0.00011378	0.0020595
BMSK0012738	26	12	12	32	48	58	31.3186667	-1.66107501	0.55022848	-3.01888227	0.00253709	0.02795396
BMSK0014965	41	28	18	77	99	69	55.558404	-1.663482412	0.42718228	-3.89408104	9.86E-05	0.00182038
BMSK0009951	360	302	274	815	1441	514	613.932386	-1.673513759	0.27766136	-6.02717553	1.67E-09	8.27E-08
BMSK0007755	15	13	20	32	43	65	31.9751941	-1.682251322	0.56103146	-2.9984973	0.00271315	0.02936184
BMSK0014368	30	25	20	70	107	46	49.671061	-1.701148043	0.46196675	-3.68240365	0.00023105	0.00385343
BMSK0002415	59	46	38	151	150	114	94.6553447	-1.704106759	0.34601002	-4.92502135	8.44E-07	2.61E-05
BMSK0005827	2767	1854	1600	5542	6333	6182	4096.37833	-1.715270836	0.17926448	-9.5683808	1.09E-21	1.71E-19
BMSK0008347	414	333	363	907	1467	991	748.720469	-1.725348794	0.20006791	-8.62381567	6.48E-18	7.29E-16
BMSK0002578	1541	926	1132	1605	3643	5484	2395.74024	-1.735487217	0.29718336	-5.83978591	5.23E-09	2.46E-07
BMSK0013201	24	12	9	57	42	29	29.3563256	-1.735976749	0.59154582	-2.93464458	0.0033393	0.03489625
BMSK0011447	42	29	7	88	89	51	51.4496642	-1.746866885	0.5338829	-3.27200382	0.00106788	0.01387789
BMSK0002364	24	24	4	46	40	68	35.2717059	-1.74787535	0.61792671	-2.8286127	0.00467502	0.04542092
BMSK0013184	34	6	10	50	46	44	31.5876417	-1.77017554	0.60120414	-2.94438349	0.00323599	0.03408997
BMSK0001142	47	53	58	166	210	135	113.481496	-1.806104795	0.3387824	-5.33116473	9.76E-08	3.63E-06
BMSK0014193	121688	62097	126112	250157	534251	222551	216625.931	-1.819848631	0.2631325	-6.91609223	4.64E-12	3.31E-10
BMSK0006512	153	106	59	338	263	372	220.731681	-1.822439421	0.32145536	-5.669339	1.43E-08	6.23E-07
BMSK0009657	82	29	41	147	239	87	102.604963	-1.824518336	0.39854346	-4.57796579	4.70E-06	0.00012344
BMSK0013326	30	8	5	41	52	36	28.3222423	-1.844820847	0.63223722	-2.91792508	0.00352369	0.03640614
BMSK0004449	46	14	16	52	93	91	51.4242809	-1.854943813	0.47965986	-3.86720671	0.00011009	0.00200468
BMSK0002808	55	30	32	192	76	88	82.4734165	-1.855586321	0.456965	-4.06067495	4.89E-05	0.00100089
BMSK0006531	416	112	258	718	1028	710	537.547004	-1.858051167	0.28526918	-6.51332602	7.35E-11	4.53E-09
BMSK0012752	14	10	6	32	22	42	21.6813942	-1.885493713	0.66966347	-2.81558393	0.00486887	0.04659934
BMSK0008711	25	65	51	72	253	194	110.405368	-1.889152969	0.46514873	-4.06139553	4.88E-05	0.00100005
BMSK0013129	12	20	10	44	49	51	31.9487225	-1.88923371	0.56456822	-3.3463338	0.00081888	0.01121715
BMSK0005351	537	170	178	741	1246	826	607.89374	-1.896689609	0.28978878	-6.54507618	5.95E-11	3.72E-09
BMSK0008004	24	10	15	79	48	29	35.1938107	-1.904627745	0.57859249	-3.29182933	0.00099538	0.01312489
BMSK0006798	144	45	79	203	429	254	189.020062	-1.909780465	0.32686643	-5.84269374	5.14E-09	2.43E-07
BMSK0011494	7244	4600	4386	22609	18794	11828	11844.9504	-1.909818255	0.23883453	-7.99640757	1.28E-15	1.27E-13
BMSK0012556	50	20	13	104	111	54	58.757471	-1.92721292	0.48569589	-3.96794157	7.25E-05	0.00139827
BMSK0009850	149	52	96	282	388	331	216.897937	-1.95259824	0.28608373	-6.82526834	8.78E-12	6.07E-10
BMSK0009146	100	28	54	170	197	235	131.591707	-1.958240902	0.35353996	-5.53895214	3.04E-08	1.23E-06
BMSK0000966	2	25	27	67	82	59	45.3437524	-1.960972796	0.64562727	-3.03731408	0.00238697	0.0265623
BMSK0015697	32	27	34	137	110	77	71.7446755	-1.962251211	0.41372628	-4.74287298	2.11E-06	6.01E-05
BMSK0002398	15	7	15	37	37	53	27.9583502	-1.964831776	0.58836561	-3.33947422	0.00083937	0.0114122
BMSK0009375	156	113	197	622	324	653	360.579063	-1.968881199	0.33152073	-5.93893847	2.87E-09	1.39E-07
BMSK0015956	46	22	18	130	79	77	63.8796627	-1.978262222	0.45609398	-4.33740041	1.44E-05	0.000339
BMSK0005642	22	18	8	49	67	54	36.705955	-1.983342981	0.5234025	-3.78932652	0.00015106	0.00264001
BMSK0002960	87	64	95	458	191	198	192.259684	-1.992035768	0.38852817	-5.12713344	2.94E-07	1.01E-05
BMSK0002846	5483	5935	4346	28176	13949	13370	12503.6002	-1.994215835	0.52315413	-3.81190882	0.0001379	0.00244755
BMSK0005405	6	16	6	27	48	33	23.0894865	-1.999038378	0.68182022	-2.93191419	0.0033688	0.03516424
BMSK0003534	22	16	6	54	67	36	33.7525537	-2.007349932	0.56862402	-3.53018842	0.00041526	0.00639941
BMSK0009441	2055	1802	1209	5370	7735	5565	3997.88133	-2.017550447	0.20369219	-9.9048982	3.96E-23	7.09E-21
BMSK0007465	245	143	179	530	904	637	440.216187	-2.02002505	0.2193682	-9.20837685	3.31E-20	4.58E-18
BMSK0002020	420	352	271	1709	1232	859	835.505586	-2.046303305	0.27584943	-7.41818937	1.19E-13	1.04E-11
BMSK0002787	248	118	140	390	506	916	392.486645	-2.04883476	0.30932847	-6.6234923	3.51E-11	2.25E-09
BMSK0008094	62	12	9	83	79	114	59.9707182	-2.050333715	0.54335204	-3.77349043	0.00016098	0.00279204
BMSK0006626	25	21	11	65	57	87	45.6843152	-2.058144412	0.49456921	-4.16148915	3.16E-05	0.00067771
BMSK0010102	25	35	18	91	98	110	64.7859602	-2.064822194	0.43236246	-4.77567405	1.79E-06	5.24E-05
BMSK0004547	106	56	42	389	139	183	160.305648	-2.075203313	0.42109451	-4.92811776	8.30E-07	2.59E-05
BMSK0007633	12	20	18	80	21	87	42.5902412	-2.077530335	0.61846983	-3.35914579	0.00078184	0.01079079
BMSK0002387	52	26	12	202	68	40	70.1591045	-2.083656621	0.58967343	-3.53357725	0.00040998	0.00633934
BMSK0014037	104	110	57	327	436	315	228.542464	-2.121271989	0.29438064	-7.20588155	5.77E-13	4.66E-11
BMSK0013080	28	4	11	38	60	62	33.6132107	-2.144412727	0.5951612	-3.60307883	0.00031447	0.00505981
BMSK0009151	69	40	40	212	199	182	126.590081	-2.186576072	0.31647724	-6.9091101	4.88E-12	3.45E-10
BMSK0013427	43	9	17	82	114	82	57.6919093	-2.245227925	0.47005242	-4.77654796	1.78E-06	5.23E-05
BMSK0007210	3899	6494	4201	20603	23342	21484	13764.6844	-2.251449599	0.25920871	-8.6858564	3.76E-18	4.29E-16
BMSK0005375	20	44	10	54	126	161	70.5796798	-2.263758609	0.54183931	-4.17791506	2.94E-05	0.00064209
BMSK0003392	472	400	365	1610	2465	1410	1130.67723	-2.273283353	0.20808007	-10.9250413	8.75E-28	1.95E-25
BMSK0013626	18	6	10	44	52	48	30.0291426	-2.28955792	0.56808618	-4.03030035	5.57E-05	0.00111947
BMSK0004756	260	124	286	517	1268	1221	613.191583	-2.302507194	0.30544699	-7.53815633	4.77E-14	4.31E-12
BMSK0004884	13	11	6	35	42	57	28.0112445	-2.32672907	0.59941753	-3.88165003	0.00010375	0.00190063
BMSK0010931	64	10	32	113	180	164	93.6625751	-2.344401231	0.4320825	-5.4258185	5.77E-08	2.22E-06
BMSK0011062	8	2	17	55	19	45	25.886398	-2.356189063	0.74782259	-3.15073267	0.00162861	0.01939592
BMSK0004115	892	696	474	2418	3415	3772	1979.73767	-2.378395362	0.2222321	-10.7023036	9.93E-27	2.11E-24
BMSK0001549	61	42	64	237	263	278	161.664945	-2.378699609	0.2947844	-8.06928602	7.07E-16	7.33E-14
BMSK0002842	10	6	11	37	46	43	26.032887	-2.378778442	0.60224867	-3.94982763	7.82E-05	0.00148643
BMSK0007774	14	10	12	54	76	40	34.7167854	-2.380143268	0.53483656	-4.45022542	8.58E-06	0.00021266
BMSK0007077	14	6	4	16	64	35	22.5143649	-2.411864048	0.70920275	-3.40081036	0.00067186	0.00950297
BMSK0009881	17	7	8	61	20	61	30.6157824	-2.421115737	0.65184176	-3.71426913	0.00020379	0.00346864
BMSK0009563	20	0	10	16	72	50	27.0516093	-2.42135504	0.78441109	-3.08684446	0.00202293	0.02306904
BMSK0009079	648	400	497	1968	1458	3831	1528.04619	-2.437027138	0.30228922	-8.06190556	7.51E-16	7.61E-14
BMSK0010714	18	20	30	67	140	138	69.7733874	-2.438427157	0.44208601	-5.51573018	3.47E-08	1.40E-06
BMSK0009862	30	28	19	183	81	100	77.9384564	-2.445439209	0.45980947	-5.31837501	1.05E-07	3.87E-06
BMSK0001853	130	32	42	354	282	280	190.841023	-2.450855127	0.36632746	-6.69033957	2.23E-11	1.47E-09
BMSK0015161	136	66	134	268	702	698	334.300701	-2.451512284	0.32442712	-7.55643442	4.14E-14	3.82E-12
BMSK0003455	12	16	9	47	94	51	38.3685451	-2.46493927	0.53623083	-4.59678763	4.29E-06	0.00011412
BMSK0014420	167	85	130	664	479	750	394.368108	-2.521819674	0.27913263	-9.03448542	1.65E-19	2.21E-17
BMSK0014124	47	14	30	108	169	179	92.0030245	-2.524277008	0.39113388	-6.45374169	1.09E-10	6.46E-09
BMSK0002362	18	26	1	135	59	34	48.5389045	-2.525517105	0.71165269	-3.5488057	0.00038698	0.00606605

BMSK0004541	12	4	1	21	19	41	16.7072148	-2.529664661	0.8402326	-3.0106719	0.0026067	0.02848019
BMSK0010165	112	86	127	333	766	650	347.500967	-2.538113159	0.28455884	-8.91946685	4.69E-19	5.94E-17
BMSK0005042	3	4	12	32	57	18	21.1441911	-2.553881855	0.76336929	-3.34553915	0.00082123	0.01123247
BMSK0005833	4	0	12	16	56	18	17.2535387	-2.559402188	0.91807345	-2.78779675	0.00530678	0.0494606
BMSK0010417	146	84	65	541	449	525	311.779038	-2.574843681	0.27699375	-9.2956744	1.46E-20	2.09E-18
BMSK0013866	87	68	75	465	384	371	250.926435	-2.579883647	0.2727277	-9.45955852	3.09E-21	4.70E-19
BMSK0013357	139	46	65	416	235	612	263.00775	-2.611892414	0.37483088	-6.96818896	3.21E-12	2.38E-10
BMSK0000961	0	17	2	40	24	51	24.0276536	-2.612337483	0.9080613	-2.87682945	0.00401693	0.04022659
BMSK0005040	22	6	10	74	52	69	40.1598923	-2.613252922	0.54403878	-4.80343136	1.56E-06	4.65E-05
BMSK0011406	4	2	4	16	28	12	11.0122833	-2.620187537	0.925702	-2.83048706	0.00464772	0.04520378
BMSK0007066	32	12	14	113	113	82	62.2779701	-2.626244406	0.44068637	-5.95944099	2.53E-09	1.24E-07
BMSK0013952	7	4	7	29	43	31	20.4534673	-2.664745638	0.68484438	-3.8910236	9.98E-05	0.00183975
BMSK0011266	6	8	0	14	34	35	16.3285406	-2.680815794	0.88374719	-3.03346456	0.00241763	0.02683218
BMSK0003084	24	17	21	20	235	128	71.70443	-2.688661182	0.57903556	-4.64334382	3.43E-06	9.34E-05
BMSK0011471	12	2	4	27	53	22	19.6998296	-2.710677132	0.75345432	-3.59766618	0.00032109	0.00514809
BMSK0009905	21	4	10	92	50	47	38.9421147	-2.713872262	0.59550222	-4.55728317	5.18E-06	0.00013469
BMSK0004409	35	4	24	112	129	113	70.612837	-2.723592664	0.47908729	-5.68496123	1.31E-08	5.71E-07
BMSK0009286	18	14	2	58	95	52	40.0560601	-2.750751937	0.5886145	-4.67326568	2.96E-06	8.17E-05
BMSK0009241	106	97	152	306	1462	587	440.619356	-2.763022819	0.59578693	-4.6376023	3.52E-06	9.53E-05
BMSK0014792	0	6	4	34	24	9	13.632294	-2.795308794	1.00002542	-2.79523774	0.00518616	0.0487766
BMSK0013721	6	14	2	66	34	40	28.8340098	-2.808561892	0.71088973	-3.9507701	7.79E-05	0.00148368
BMSK0009397	218	136	92	805	1042	916	545.410912	-2.813778808	0.23885224	-11.7804163	4.92E-32	1.45E-29
BMSK0013101	2	0	8	10	28	28	12.7593747	-2.822009387	1.00877541	-2.79746051	0.00515061	0.04849224
BMSK0016011	6	4	0	16	29	19	12.3650133	-2.852879041	0.9502043	-3.00238491	0.00267873	0.02905835
BMSK0004821	30	23	27	240	174	104	104.14643	-2.873570606	0.40719737	-7.05694791	1.70E-12	1.31E-10
BMSK0004544	20	4	0	38	33	69	27.9799384	-2.87890711	0.78645286	-3.66062258	0.0002516	0.00412837
BMSK0010293	19	17	9	99	84	110	58.6753287	-2.882287826	0.46160711	-6.24402829	4.26E-10	2.42E-08
BMSK0003865	2	12	6	38	48	56	28.1257923	-2.883314529	0.68624728	-4.2015679	2.65E-05	0.00058412
BMSK0004542	3	5	1	22	13	25	12.1948234	-2.899381993	0.94775762	-3.05920199	0.00221927	0.02505748
BMSK0012237	35	16	27	132	208	183	101.617782	-2.911669698	0.35625987	-8.1728816	3.01E-16	3.19E-14
BMSK0001873	153	54	71	134	861	916	360.338905	-2.959922987	0.7571604	-3.90924166	9.26E-05	0.00173087
BMSK0013762	17	2	10	91	62	35	37.5232707	-2.961682573	0.63335043	-4.67621464	2.92E-06	8.08E-05
BMSK0003598	1253	690	1262	6170	10654	5969	4367.39312	-2.965876865	0.22416873	-13.2305557	5.85E-40	2.81E-37
BMSK0014371	26	6	14	94	117	102	60.8907356	-2.981677555	0.45758909	-6.51605904	7.22E-11	4.48E-09
BMSK0003095	10	0	1	26	36	9	13.506174	-3.000056206	1.02590052	-2.92431493	0.00345215	0.03582934
BMSK0010190	20	8	11	11	141	138	53.824901	-3.034781756	0.66102426	-4.59102931	4.41E-06	0.00011697
BMSK0011949	6	7	2	94	2	5	21.9518273	-3.04467527	1.07660395	-2.82803651	0.00468345	0.04542653
BMSK0007289	45	21	14	274	185	99	110.872205	-3.047372382	0.44815324	-6.79984451	1.05E-11	7.13E-10
BMSK0009288	8	8	10	42	125	39	38.0741307	-3.05063866	0.59676439	-5.11196495	3.19E-07	1.09E-05
BMSK0009296	19	4	13	51	155	66	50.3076858	-3.066050675	0.54338547	-5.64249665	1.68E-08	7.08E-07
BMSK0015808	79	44	52	364	459	484	253.383999	-3.078832242	0.26122304	-11.7862202	4.60E-32	1.40E-29
BMSK0006405	350	135	135	1636	1273	1478	866.769501	-3.079800721	0.2726732	-11.294842	1.39E-29	3.63E-27
BMSK0015122	110	14	22	238	401	370	192.939855	-3.083848576	0.43862703	-7.03068517	2.06E-12	1.56E-10
BMSK0006130	730	440	348	3444	3235	4844	2256.98417	-3.133024066	0.23997542	-13.0556039	5.90E-39	2.45E-36
BMSK0009866	59	48	45	553	307	322	235.598281	-3.156883807	0.3360907	-9.39295194	5.83E-21	8.58E-19
BMSK0002377	1444	1074	1324	7467	12169	11928	6021.21726	-3.174282647	0.19229227	-16.5075932	3.24E-61	2.68E-58
BMSK0009595	101	61	68	456	815	612	356.196792	-3.18272768	0.24324144	-13.0846441	4.03E-39	1.75E-36
BMSK0002577	3	4	5	28	41	38	20.3777405	-3.272700528	0.73502181	-4.45252163	8.49E-06	0.00021097
BMSK0009690	12	0	6	75	39	27	27.8517357	-3.279169606	0.76763018	-4.27180915	1.94E-05	0.00044333
BMSK0009907	200	78	139	480	1777	1490	689.764388	-3.318792093	0.33362783	-9.94758779	2.58E-23	4.71E-21
BMSK0015107	4	2	0	2	36	21	10.4155029	-3.427524815	1.18895508	-2.88280429	0.00394152	0.0396893
BMSK0007184	4	6	6	43	68	53	30.7043135	-3.464909691	0.62312017	-5.5605802	2.69E-08	1.10E-06
BMSK0010678	4	0	2	18	13	24	10.6042904	-3.466671095	1.06686087	-3.24941254	0.00115644	0.01476317
BMSK0011830	2	2	6	0	54	54	19.3918559	-3.471672625	1.07353082	-3.23388257	0.0012212	0.01535597
BMSK0009954	40	34	55	376	496	445	248.061392	-3.48410296	0.28436129	-12.2523814	1.63E-34	5.51E-32
BMSK0000875	2	3	0	16	4	29	9.72953982	-3.495687682	1.20332749	-2.90501773	0.00367232	0.03747495
BMSK0009992	0	6	8	50	64	46	30.054305	-3.564859055	0.71908658	-4.95748239	7.14E-07	2.24E-05
BMSK0005238	3	4	2	44	16	35	18.7216921	-3.598276156	0.84736819	-4.24641404	2.17E-05	0.00048452
BMSK0013062	0	4	2	12	42	22	13.6524449	-3.652646915	1.01102653	-3.61281014	0.0003029	0.00489951
BMSK0001010	2	0	4	7	38	26	12.6408992	-3.665380023	1.05535529	-3.47312422	0.00051444	0.00766864
BMSK0009961	18	0	8	105	75	99	53.1015983	-3.717410586	0.59949369	-6.20091693	5.61E-10	3.07E-08
BMSK0012286	6	0	3	16	50	40	19.0398943	-3.752427943	0.86432835	-4.34143799	1.42E-05	0.00033456
BMSK0011753	20	9	8	140	161	142	82.4412134	-3.777079052	0.41890255	-9.01660545	1.94E-19	2.57E-17
BMSK0010868	8	0	6	36	65	67	30.7901274	-3.786966471	0.69506919	-5.4483302	5.08E-08	2.01E-06
BMSK0000466	2	1	0	19	10	7	6.90185074	-3.839044957	1.36415452	-2.81423029	0.00488942	0.04661042
BMSK0010329	2	6	0	24	44	41	19.9878998	-3.849114891	0.86234432	-4.46354757	8.06E-06	0.00020204
BMSK0009874	1	0	2	6	20	14	7.13828365	-3.852443486	1.33591582	-2.88374718	0.00392974	0.0396144
BMSK0014589	102	24	64	793	919	648	436.748895	-3.855254551	0.30143216	-12.7897916	1.87E-37	7.11E-35
BMSK0003071	4	0	4	24	36	42	18.7838354	-3.864762248	0.85161556	-4.53815361	5.67E-06	0.00014584
BMSK0009693	5	3	4	101	41	13	30.0675046	-3.924592914	0.80468337	-4.87718902	1.08E-06	3.29E-05
BMSK0001960	75	53	29	55	859	1258	388.921664	-3.925362969	1.05899069	-3.70670204	0.00020998	0.00354742
BMSK0016067	188	108	76	1433	1546	2008	927.839984	-3.950570463	0.25349567	-15.5843708	9.30E-55	5.65E-52
BMSK0007474	4	0	0	10	14	27	9.39223484	-3.980962438	1.25267376	-3.17797225	0.00148309	0.01799232
BMSK0008492	2	0	2	28	18	8	10.1898308	-3.984322007	1.17447132	-3.39243875	0.00069273	0.00973777
BMSK0009043	6	30	12	177	289	275	135.478012	-3.988184013	0.45897983	-8.68923586	3.65E-18	4.21E-16
BMSK0010357	40	40	14	472	402	460	249.675967	-4.002102541	0.33895295	-11.8072508	3.58E-32	1.13E-29
BMSK0011749	4	0	0	24	8	18	9.61771754	-4.013878696	1.26165679	-3.1814347	0.00146548	0.01784847
BMSK0001599	10	5	9	126	156	66	63.5101024	-4.015475789	0.5114491	-7.85117388	4.12E-15	4.00E-13
BMSK0007955	6	2	0	20	82	16	20.1769542	-4.026896207	0.93024324	-4.32886373	1.50E-05	0.00035151
BMSK0001600	120	27	58	1548	636	494	516.184996	-4.029212536	0.71962683	-5.59903048	2.16E-08	8.98E-07
BMSK0003572	55	44	50	67	1430	1000	429.880594	-4.120854008	0.98884134	-4.1673561	3.08E-05	0.00066304
BMSK0006966	40	18	18	255	599	360	215.43321	-4.151064231	0.33769805	-12.2922363	9.97E-35	3.50E-32
BMSK0012362	10	4	0	24	109	95	40.0335656	-4.206246452	0.72658028	-5.78910077	7.08E-09	3.21E-07
BMSK0014444	2	4	0	12	47	47	18.7976666	-4.226742136	0.94794976	-4.45882506	8.24E-06	0.00020598
BMSK0012039	36	24	20	428	130	735	247.19804	-4.256462839	0.75480769	-5.63913547	1.71E-08	7.19E-07
BMSK0003940	2	0	6	18	66	59	25.2358718	-4.263909926	0.84700894	-5.03407903	4.80E-07	1.54E-05
BMSK0009660	2	0	4	28	68	12	18.7294841	-4.267270284	0.96730454	-4.4115065	1.03E-05	0.00024974
BMSK0015666	48	14	22	342	816	330	260.494013	-4.312139423	0.36152366	-11.9276825	8.49E-33	2.77E-30
BMSK0010131	62	22	10	343	876	436	289.890385	-4.330398844	0.39127838	-11.0673092	1.81E-28	4.23E-26
BMSK0001413	4	0	0	35	16	14	12.2493837	-4.365109204	1.17754499	-3.70695747	0.00020976	0.00354742

BMSK0007486	7	4	0	102	74	22	36.5817167	-4.401036458	0.77405883	-5.68566143	1.30E-08	5.71E-07
BMSK0009096	0	0	2	8	23	10	7.13642858	-4.412697111	1.4783467	-2.9848865	0.00283684	0.03059155
BMSK0015909	28	0	5	123	92	311	97.9197464	-4.412782106	1.39305431	-3.16770284	0.00153648	0.01849255
BMSK0009536	2	0	0	11	8	16	6.46720537	-4.429019543	1.53828874	-2.87918608	0.00398703	0.03997107
BMSK0004054	0	0	2	12	15	13	7.25840547	-4.435130001	1.45376804	-3.05078243	0.00228246	0.02564393
BMSK0010759	0	0	2	8	12	20	7.28196465	-4.438408783	1.47276088	-3.01366558	0.00258112	0.02830237
BMSK0006689	0	3	0	15	23	25	11.4119429	-4.450521583	1.20147592	-3.70421204	0.00021205	0.00356923
BMSK0009042	8	10	7	123	221	165	90.7411216	-4.465307717	0.44422646	-10.0518724	9.01E-24	1.71E-21
BMSK0001423	6	2	9	12	291	80	62.8917642	-4.518793951	1.17836367	-3.83480421	0.00012566	0.00226121
BMSK0009676	34	18	39	841	311	702	350.968403	-4.575841773	0.39193532	-11.674992	1.71E-31	4.88E-29
BMSK0015450	0	0	2	16	21	8	8.04261972	-4.592252857	1.43640142	-3.19705398	0.00138839	0.01711659
BMSK0009995	3	0	2	75	16	6	18.8466547	-4.601947369	1.1109334	-4.14241517	3.44E-05	0.00073254
BMSK0009932	32	36	46	611	832	1104	460.32315	-4.607240449	0.2992043	-15.3983094	1.68E-53	9.58E-51
BMSK0002580	0	4	4	71	47	63	33.622815	-4.621340414	0.7523465	-6.14256916	8.12E-10	4.23E-08
BMSK0013788	12	8	0	124	128	196	81.5255047	-4.6938432	0.53852841	-8.71605501	2.88E-18	3.41E-16
BMSK0013720	0	2	0	18	18	21	10.348342	-4.934822535	1.33997295	-3.68277772	0.00023071	0.00385343
BMSK0012640	1	0	0	5	17	10	5.44104732	-4.982877128	1.67844745	-2.96874181	0.00299022	0.03209383
BMSK0013989	4	2	0	72	64	37	31.1635987	-5.057431315	0.81814131	-6.18161102	6.35E-10	3.43E-08
BMSK0009057	2	0	0	22	33	3	10.0310679	-5.078067921	1.47227063	-3.44914028	0.00056237	0.00814372
BMSK0005228	26	27	18	805	661	742	400.647857	-5.12921287	0.30198816	-16.9848147	1.06E-64	9.71E-62
BMSK0013763	12	15	22	562	568	508	293.966424	-5.203787914	0.3284497	-15.8434849	1.56E-56	1.02E-53
BMSK0014683	0	1	2	39	30	41	19.9877854	-5.343725034	1.05192783	-5.07993505	3.78E-07	1.24E-05
BMSK0001558	202	186	187	6476	3737	10476	3794.7913	-5.349138473	0.50233198	-10.6486122	1.77E-26	3.67E-24
BMSK0011814	0	0	0	11	8	2	3.70729491	-5.416812763	1.93569046	-2.7983879	0.00513584	0.04840316
BMSK0008724	18	18	18	808	627	758	396.35164	-5.516516282	0.3196556	-17.2576867	9.80E-67	9.93E-64
BMSK0010704	0	0	0	12	4	6	4.02093437	-5.530333265	1.87329803	-2.95219082	0.00315528	0.03344743
BMSK0001711	0	0	0	8	14	2	4.01917302	-5.539238399	1.89276674	-2.92652986	0.00342767	0.03569703
BMSK0007002	174	135	118	8818	103	7578	3164.54445	-5.543238355	1.35870364	-4.07979944	4.51E-05	0.00093035
BMSK0015125	0	0	0	6	12	6	4.04571659	-5.548506764	1.83896701	-3.01718668	0.00255133	0.02805229
BMSK0013867	0	0	0	1	13	11	4.11417923	-5.575231305	1.91645554	-2.90913678	0.00362428	0.03719272
BMSK0015642	0	0	0	4	18	4	4.19457942	-5.605374913	1.86302528	-3.00874871	0.00262326	0.02859082
BMSK0007168	4	0	2	65	68	124	45.9571278	-5.63797466	0.7731963	-7.29177654	3.06E-13	2.56E-11
BMSK0011315	1	0	0	31	16	0	8.58230768	-5.644368399	1.73260521	-3.25773487	0.00112305	0.0143899
BMSK0012105	2	0	0	20	43	26	15.2606913	-5.660869551	1.27839813	-4.42809593	9.51E-06	0.00023315
BMSK0009224	12	0	0	162	182	109	79.9700434	-5.66266468	1.63310364	-3.46742517	0.00052547	0.00773204
BMSK0008247	0	0	1	0	23	32	9.34848346	-5.768932521	1.69416736	-3.40517275	0.00066122	0.00936697
BMSK0004543	5	0	0	53	43	129	40.5532286	-5.772285887	0.89352971	-6.46009393	1.05E-10	6.24E-09
BMSK0002407	22	10	0	270	743	507	259.537528	-5.784553639	1.16028527	-4.98545814	6.18E-07	1.96E-05
BMSK0015888	0	0	0	18	3	5	4.86508521	-5.803501957	1.8294189	-3.17231989	0.00151226	0.01829758
BMSK0003879	0	0	0	4	14	11	4.85050563	-5.811312571	1.76517145	-3.29220856	0.00099404	0.01312489
BMSK0014496	53	13	14	1349	1677	974	700.869641	-5.871963829	0.32404478	-18.1208409	2.18E-73	2.49E-70
BMSK0013907	0	0	0	11	11	8	5.23215418	-5.915392209	1.71132251	-3.45662036	0.00054699	0.00794623
BMSK0001462	0	0	0	4	17	12	5.4790076	-5.987849984	1.71839222	-3.48456536	0.00049294	0.00740867
BMSK0014006	0	0	0	8	7	17	5.66004041	-6.027709113	1.70038506	-3.5449083	0.00039275	0.00611979
BMSK0005700	0	0	0	21	2	8	5.83973898	-6.067012543	1.77432207	-3.4193412	0.00062773	0.00893413
BMSK0014587	0	0	0	6	10	19	6.07675857	-6.132348451	1.67473772	-3.66167691	0.00025057	0.00411882
BMSK0009878	0	0	0	2	33	4	6.0496767	-6.135983638	1.78693536	-3.4338028	0.00059518	0.00851066
BMSK0004737	0	0	0	24	3	7	6.39679393	-6.198515184	1.73145021	-3.57995577	0.00034365	0.00548101
BMSK0009577	0	0	0	14	2	20	6.62230307	-6.251016059	1.71540726	-3.64404198	0.00026839	0.00438018
BMSK0014620	0	2	8	182	290	272	129.483812	-6.266066258	0.96658873	-6.48266014	9.01E-11	5.52E-09
BMSK0013919	8	6	8	461	564	545	275.744812	-6.31653901	0.38745057	-16.3028256	9.42E-60	6.61E-57
BMSK0005743	0	0	0	26	10	2	6.93969641	-6.318764424	1.71491311	-3.68459742	0.00022906	0.00383441
BMSK0010362	0	0	0	16	17	8	7.10825336	-6.358095807	1.6008817	-3.97162126	7.14E-05	0.00138563
BMSK0008560	14	0	8	1113	51	304	282.850038	-6.488364322	1.62772244	-3.98616138	6.72E-05	0.00131181
BMSK0004151	0	0	1	52	23	14	16.2890341	-6.573622841	1.42184194	-4.62331478	3.78E-06	0.00010104
BMSK0012354	0	0	0	4	22	25	8.5582541	-6.629829435	1.58651101	-4.17887388	2.93E-05	0.00064092
BMSK0013759	0	0	0	17	19	15	8.85816822	-6.6755473	1.51695957	-4.4006099	1.08E-05	0.00025984
BMSK0008716	1	0	0	51	7	38	17.9574843	-6.718329665	1.44334312	-4.65470032	3.24E-06	8.86E-05
BMSK0003983	0	0	0	7	48	7	9.81128178	-6.83168533	1.57896647	-4.32668169	1.51E-05	0.0003541
BMSK0009888	1	0	0	42	27	42	19.9096915	-6.870382825	1.35281332	-5.078589	3.80E-07	1.25E-05
BMSK0001598	0	0	4	183	84	161	77.8935009	-6.892483393	1.26597717	-5.44439786	5.20E-08	2.04E-06
BMSK0011430	0	0	0	33	14	10	10.3415355	-6.894471198	1.52224745	-4.52913958	5.92E-06	0.00015092
BMSK0002215	0	0	0	10	32	20	10.3324976	-6.902494799	1.49246619	-4.62489191	3.75E-06	0.00010057
BMSK0010199	0	0	0	12	19	31	10.7488734	-6.955258658	1.48442698	-4.68548387	2.79E-06	7.79E-05
BMSK0006712	0	0	0	25	18	20	11.1690031	-7.007663928	1.46250708	-4.79154188	1.66E-06	4.89E-05
BMSK0013765	0	0	12	519	482	498	264.944751	-7.037110214	1.85471856	-3.79416607	0.00014814	0.00259901
BMSK0011815	0	0	0	48	9	6	11.8086467	-7.084525899	1.56616188	-4.5234953	6.08E-06	0.00015457
BMSK0008470	0	0	0	28	16	26	12.5317277	-7.172891874	1.44467194	-4.96506625	6.87E-07	2.16E-05
BMSK0011813	0	0	0	25	32	16	12.5483263	-7.179069313	1.43544893	-5.00127113	5.70E-07	1.82E-05
BMSK0010361	0	0	0	24	14	34	12.8843019	-7.213285359	1.44856652	-4.97960241	6.37E-07	2.01E-05
BMSK0003890	0	0	0	24	22	28	13.0066211	-7.228491716	1.42398219	-5.0762515	3.85E-07	1.25E-05
BMSK0004789	0	0	0	25	26	24	13.0840369	-7.237807146	1.41898892	-5.10067912	3.38E-07	1.15E-05
BMSK0003735	0	0	0	56	14	1	13.225228	-7.253220303	2.48922617	-2.91384543	0.00357007	0.03671896
BMSK0009882	11	13	4	1043	1561	1306	676.256792	-7.26913437	0.34492726	-21.074398	1.37E-98	2.08E-95
BMSK0003829	4	0	0	146	270	166	99.2370626	-7.348526171	0.80814964	-9.09302664	9.63E-20	1.31E-17
BMSK0013761	4	2	0	261	464	205	158.133328	-7.418704883	0.67517326	-10.9878535	4.37E-28	9.97E-26
BMSK0014005	0	0	0	29	31	31	15.8697652	-7.516367113	1.38120199	-5.44190289	5.27E-08	2.06E-06
BMSK0011847	37	24	34	5728	4821	6133	2956.12141	-7.640976102	0.25857963	-29.5497988	6.61E-192	3.01E-188
BMSK0011812	0	0	0	56	26	16	17.7112219	-7.671530986	1.41159737	-5.43464527	5.49E-08	2.13E-06
BMSK0003797	0	0	0	32	48	28	18.4641733	-7.737009548	1.35809959	-5.69693827	1.22E-08	5.40E-07
BMSK0015229	1	0	0	70	75	65	36.6953969	-7.759126384	1.27453983	-6.08778652	1.14E-09	5.80E-08
BMSK0008715	6	8	2	1499	427	1195	573.826559	-7.817180664	0.84049772	-9.30065656	1.40E-20	2.02E-18
BMSK0009868	0	0	2	81	183	165	73.1559826	-7.868875183	1.09459293	-7.18885984	6.53E-13	5.18E-11
BMSK0009865	0	0	0	38	48	35	20.8922393	-7.914144762	1.33714104	-5.91870606	3.24E-09	1.56E-07
BMSK0013132	83	130	114	31122	27023	15252	12919.4444	-7.920309976	0.29550922	-26.8022429	3.04E-158	9.25E-155
BMSK0004100	0	0	0	43	74	12	21.6399649	-7.967089776	1.38949108	-5.7338186	9.82E-09	4.37E-07
BMSK0009869	6	0	2	446	879	701	345.584879	-8.141358951	0.57518257	-14.1543909	1.75E-45	9.42E-43
BMSK0014926	15	23	6	4088	5244	2343	2011.72768	-8.150879797	0.62470251	-13.0476181	6.56E-39	2.60E-36
BMSK0010292	0	0	0	30	31	83	25.3874088	-8.193841683	1.35188205	-6.06106255	1.35E-09	6.78E-08
BMSK0009861	3	3	0	514	640	557	297.119555	-8.317931471	0.63440176	-13.1114571	2.83E-39	1.29E-36

BMSK0000571	0	0	0	32	62	83	30.4205126	-8.456944781	1.31164784	-6.44757268	1.14E-10	6.69E-09
BMSK0002568	0	0	0	86	18	62	30.62559	-8.461439522	1.35904442	-6.22602133	4.78E-10	2.66E-08
BMSK0016014	3	1	5	1377	557	891	513.777579	-8.517673881	0.78689978	-10.824344	2.64E-27	5.73E-25
BMSK0009864	8	10	5	2132	3062	2631	1350.72546	-8.555926349	0.35531464	-24.0798589	4.06E-128	7.42E-125
BMSK0009902	37	26	36	8768	15448	14003	6553.42016	-8.730257697	0.24543848	-35.570044	4.07E-277	3.71E-273
BMSK0003734	0	0	0	136	65	14	38.8348862	-8.807908061	1.83662469	-4.79570384	1.62E-06	4.82E-05
BMSK0009914	0	0	0	162	20	40	41.841205	-8.915186527	1.91245026	-4.66165667	3.14E-06	8.62E-05
BMSK0003699	0	0	0	167	30	32	42.8821065	-8.950628216	1.85412947	-4.82740195	1.38E-06	4.16E-05
BMSK0009091	0	0	0	131	43	67	44.0642784	-8.986822925	1.30661531	-6.87794095	6.07E-12	4.26E-10
BMSK0013909	0	0	0	51	81	131	45.5856943	-9.039707582	1.2810684	-7.05638167	1.71E-12	1.31E-10
BMSK0009854	0	0	0	66	110	112	49.4550126	-9.158006948	1.25892457	-7.27446834	3.48E-13	2.88E-11
BMSK0014588	0	0	0	89	162	96	58.870467	-9.41045233	1.24810029	-7.53982064	4.71E-14	4.29E-12
BMSK0002738	0	0	0	109	84	211	71.7172444	-9.691927204	1.26421518	-7.66635884	1.77E-14	1.66E-12
BMSK0015151	0	0	0	236	155	110	89.074625	-10.00421846	1.24790279	-8.01682513	1.09E-15	1.09E-13
BMSK0000089	0	0	0	208	156	189	97.9120401	-10.14076223	1.23118677	-8.23657503	1.77E-16	1.90E-14
BMSK0004790	0	0	0	260	187	134	102.861595	-10.21211165	1.23842666	-8.24603667	1.64E-16	1.78E-14
BMSK0001557	8	2	4	5606	1947	6980	2641.03329	-10.30392455	0.80607302	-12.7828675	2.04E-37	7.46E-35
BMSK0005075	0	0	0	153	226	301	117.718469	-10.40843777	1.22827443	-8.47403275	2.37E-17	2.61E-15
BMSK0014854	0	0	0	246	321	225	136.503541	-10.62219271	1.21457829	-8.74558093	2.22E-18	2.70E-16
BMSK0005229	0	0	0	266	354	180	137.288413	-10.63067994	1.22165583	-8.70186157	3.26E-18	3.82E-16
BMSK0009950	0	0	0	207	229	388	144.322809	-10.70175101	1.22751355	-8.71823454	2.83E-18	3.39E-16
BMSK0005586	2	0	0	972	1160	1214	581.640985	-10.88128602	1.04488295	-10.4138803	2.14E-25	4.25E-23
BMSK0009867	0	0	1	465	1435	892	465.880114	-11.43164739	1.21480683	-9.41025939	4.95E-21	7.40E-19
BMSK0010335	0	0	0	675	102	832	296.408845	-11.74058715	1.81628997	-6.46404888	1.02E-10	6.12E-09
BMSK0005884	0	0	0	634	643	521	313.650022	-11.82173155	1.20333978	-9.824101	8.87E-23	1.50E-20
BMSK0001556	0	2	0	3092	1049	3839	1450.24427	-12.10992367	1.37457513	-8.80993946	1.25E-18	1.56E-16
BMSK0009059	6	4	0	14933	13262	16912	7939.03927	-12.33104173	0.50500499	-24.4176631	1.11E-131	2.53E-128
BMSK0003800	0	0	0	2731	1010	335	745.282843	-13.07084707	1.80953673	-7.22331127	5.07E-13	4.13E-11
BMSK0001555	0	0	0	6309	2037	7827	2941.76907	-15.05170536	1.52239691	-9.88684705	4.75E-23	8.33E-21
