# Supplementary material for: BmAbl1 Regulates Silk Protein Synthesis via Glutathione Metabolism in Bombyx mori
Source: Insects. 2022 Oct 22;13(11):967. doi: 10.3390/insects13110967 (PMC9696079; doi:10.3390/insects13110967)
Supplement: Supplementary file 1 [file insects-13-00967-s001.zip › Table S5.pdf]

Table S5 GO enrichment analysis of DEGs

ID	Description	ONTOLOGY	GeneRatio	BgRatio	pvalue	p.adjust	qvalue	geneID	Count
GO:0006749	glutathione metabolic process	BP	7/54	25/1602	1.00E-05	1.00E-05	0.002556887	692678/692521/100141440/100141444/100862776/692941/100141445	7
GO:0006575	cellular modified amino acid metabolic process	BP	7/54	31/1602	4.69E-05	4.69E-05	0.005976837	692678/692521/100141440/100141444/100862776/692941/100141445	7
GO:0006790	sulfur compound metabolic process	BP	7/54	46/1602	0.000644118	0.000644118	0.054750013	692678/692521/100141440/100141444/100862776/692941/100141445	7
GO:0046903	secretion	BP	3/54	10/1602	0.003675643	0.003675643	0.234322266	100301504/100174841/101746631	3
GO:0006570	tyrosine metabolic process	BP	2/54	6/1602	0.015338684	0.015338684	0.706670931	693082/692758	2
GO:0065008	regulation of biological quality	BP	4/54	36/1602	0.030520826	0.030520826	0.706670931	100301504/100174841/692783/692564	4
GO:0019439	aromatic compound catabolic process	BP	4/54	40/1602	0.042888502	0.042888502	0.706670931	693082/100499206/692686/101744496	4
GO:0030054	cell junction	CC	3/86	18/2125	0.03363747	0.03363747	0.996158161	100301504/692783/100233162	3
GO:0005576	extracellular region	CC	13/86	191/2125	0.04011497	0.04011497	0.996158161	733005/100862800/692397/101737284/101736553/100174841/692758/101746631/692973/692564/100188965/101735732/101735420	13
GO:0016614	oxidoreductase activity, acting on CH-OH group of donors	MF	7/125	46/2665	0.004898963	0.004898963	0.495780974	100124423/732951/101746394/101738196/105842055/101741535/100302629	7
GO:0016757	glycosyltransferase activity	MF	9/125	74/2665	0.006849203	0.006849203	0.495780974	101742412/100500754/100862824/692850/100862789/101744496/100862828/732962/100862804	9
GO:0005543	phospholipid binding	MF	3/125	10/2665	0.009487855	0.009487855	0.495780974	778459/101738643/100301504	3
GO:0008289	lipid binding	MF	4/125	24/2665	0.023632741	0.023632741	0.495780974	778459/101738643/100301504/101743740	4
GO:0004521	endoribonuclease activity	MF	2/125	6/2665	0.028917868	0.028917868	0.495780974	100499531/100499206	2
GO:0046872	metal ion binding	MF	31/125	480/2665	0.031924999	0.031924999	0.495780974	101735538/692562/100852388/101738020/693082/692479/101741784/100127129/100134922/778459/100036578/733030/101742623/100301504/101740969/100379594/100862788/692758/101746631/100216496/100126178/100126160/100101183/692651/101743308/692709/692564/101745819/692863/692591/692368	31
GO:0016758	hexosyltransferase activity	MF	6/125	52/2665	0.032756584	0.032756584	0.495780974	101742412/100500754/100862789/100862828/732962/100862804	6
GO:0043169	cation binding	MF	31/125	481/2665	0.032774002	0.032774002	0.495780974	101735538/692562/100852388/101738020/693082/692479/101741784/100127129/100134922/778459/100036578/733030/101742623/100301504/101740969/100379594/100862788/692758/101746631/100216496/100126178/100126160/100101183/692651/101743308/692709/692564/101745819/692863/692591/692368	31
GO:0004364	glutathione transferase activity	MF	2/125	7/2665	0.039257027	0.039257027	0.495780974	100141440/692941	2
GO:0008081	phosphoric diester hydrolase activity	MF	2/125	7/2665	0.039257027	0.039257027	0.495780974	692531/101743953	2
GO:0016763	pentosyltransferase activity	MF	2/125	7/2665	0.039257027	0.039257027	0.495780974	692850/101744496	2
GO:0030414	peptidase inhibitor activity	MF	5/125	41/2665	0.040548974	0.040548974	0.495780974	100272179/692397/101737284/100272180/692973	5
GO:0061134	peptidase regulator activity	MF	5/125	41/2665	0.040548974	0.040548974	0.495780974	100272179/692397/101737284/100272180/692973	5
