# Supplementary material for: BmAbl1 Regulates Silk Protein Synthesis via Glutathione Metabolism in Bombyx mori
Source: Insects. 2022 Oct 22;13(11):967. doi: 10.3390/insects13110967 (PMC9696079; doi:10.3390/insects13110967)
Supplement: Supplementary file 1 [file insects-13-00967-s001.zip › Table S6.pdf]

<table>
<tr><th colspan="7">Table S6 KEGG enrichment analysis of DEGs</th></tr>
<tr><th>Term</th><th>ID</th><th>Input</th><th>Background</th><th>P_Value</th><th>Corrected P_Value</th><th>Input</th></tr>
<tr><td>Glutathione metabolism</td><td>bmor00480</td><td>10</td><td>54</td><td>2.33E-04</td><td>0.011329952</td><td>BMSK0012237|BMSK0004409|BMSK0003439|BMSK0006405|BMSK0001869|BMSK0006071|BMSK0003983|BMSK0003984|BMSK0011959|BMSK0003598</td></tr>
<tr><td>Drug metabolism - other enzymes</td><td>bmor00983</td><td>13</td><td>93</td><td>0.00034925</td><td>0.011329952</td><td>BMSK0003983|BMSK0007289|BMSK0003439|BMSK0016013|BMSK0015552|BMSK0009882|BMSK0003961|BMSK0016014|BMSK0015906|BMSK0012237|BMSK0003984|BMSK0016011|BMSK0003598</td></tr>
<tr><td>Drug metabolism - cytochrome P450</td><td>bmor00982</td><td>10</td><td>58</td><td>0.00038625</td><td>0.011329952</td><td>BMSK0003983|BMSK0007289|BMSK0003439|BMSK0003598|BMSK0003961|BMSK0016014|BMSK0012237|BMSK0003984|BMSK0016011|BMSK0016013</td></tr>
<tr><td>Metabolism of xenobiotics by cytochrome P4</td><td>bmor00980</td><td>10</td><td>64</td><td>0.0007679</td><td>0.016893788</td><td>BMSK0003983|BMSK0007289|BMSK0003439|BMSK0003598|BMSK0003961|BMSK0016014|BMSK0012237|BMSK0003984|BMSK0016011|BMSK0016013</td></tr>
<tr><td>Tyrosine metabolism</td><td>bmor00350</td><td>7</td><td>34</td><td>0.00117191</td><td>0.020625572</td><td>BMSK0001189|BMSK0012908|BMSK0012909|BMSK0005604|BMSK0002040|BMSK0004278|BMSK0004276</td></tr>
<tr><td>Metabolic pathways</td><td>bmor01100</td><td>65</td><td>1142</td><td>0.01095002</td><td>0.160600234</td><td>BMSK0009961|BMSK0010319|BMSK0016048|BMSK0004409|BMSK0003439|BMSK0013883|BMSK0013101|BMSK0014113|BMSK0002040|BMSK0006405|BMSK0009882|BMSK0005067|BMSK0015906|BMSK0004276|BMSK0012237|BMSK0002846|BMSK0003598|BMSK0013141|BMSK0001495|BMSK0007275|BMSK0009445|BMSK0002496|BMSK0001159|BMSK0009441|BMSK0007486|BMSK0014693|BMSK0005586|BMSK0005850|BMSK0009950|BMSK0007289|BMSK0001494|BMSK0011062|BMSK0012908|BMSK0012909|BMSK0015552|BMSK0011266|BMSK0005604|BMSK0015169|BMSK0003961|BMSK0016014|BMSK0001125|BMSK0001126|BMSK0004278|BMSK0016011|BMSK0016013|BMSK0007184|BMSK0007523|BMSK0009224|BMSK0004631|BMSK0001189|BMSK0009430|BMSK0012277|BMSK0004756|BMSK0003392|BMSK0000579|BMSK0011000|BMSK0006071|BMSK0008722|BMSK0003863|BMSK0001869|BMSK0007210|BMSK0003983|BMSK0003984|BMSK0003840|BMSK0011959</td></tr>
<tr><td>Phenylalanine metabolism</td><td>bmor00360</td><td>3</td><td>11</td><td>0.01724719</td><td>0.216812673</td><td>BMSK0004276|BMSK0002040|BMSK0004278</td></tr>
<tr><td>Retinol metabolism</td><td>bmor00830</td><td>6</td><td>47</td><td>0.01971024</td><td>0.216812673</td><td>BMSK0007289|BMSK0001392|BMSK0003961|BMSK0016014|BMSK0016011|BMSK0016013</td></tr>
<tr><td>Ascorbate and aldarate metabolism</td><td>bmor00053</td><td>6</td><td>52</td><td>0.02937897</td><td>0.279206284</td><td>BMSK0007289|BMSK0009224|BMSK0003961|BMSK0016014|BMSK0016011|BMSK0016013</td></tr>
<tr><td>Glycine, serine and threonine metabolism</td><td>bmor00260</td><td>5</td><td>39</td><td>0.03172799</td><td>0.279206284</td><td>BMSK0009961|BMSK0001494|BMSK0009950|BMSK0001495|BMSK0014693</td></tr>
<tr><td>Phototransduction - fly</td><td>bmor04745</td><td>4</td><td>28</td><td>0.03906906</td><td>0.312552515</td><td>BMSK0004449|BMSK0009907|BMSK0009902|BMSK0009901</td></tr>
<tr><td>Biosynthesis of amino acids</td><td>bmor01230</td><td>7</td><td>75</td><td>0.04826475</td><td>0.353941506</td><td>BMSK0005586|BMSK0007210|BMSK0003392|BMSK0013101|BMSK0005067|BMSK0013141|BMSK0014693</td></tr>
</table>
