# Supplementary figures and images for: BmAbl1 Regulates Silk Protein Synthesis via Glutathione Metabolism in Bombyx mori
Source: Insects. 2022 Oct 22;13(11):967. doi: 10.3390/insects13110967 (PMC9696079; doi:10.3390/insects13110967)

## GLUTATHIONE METABOLISM

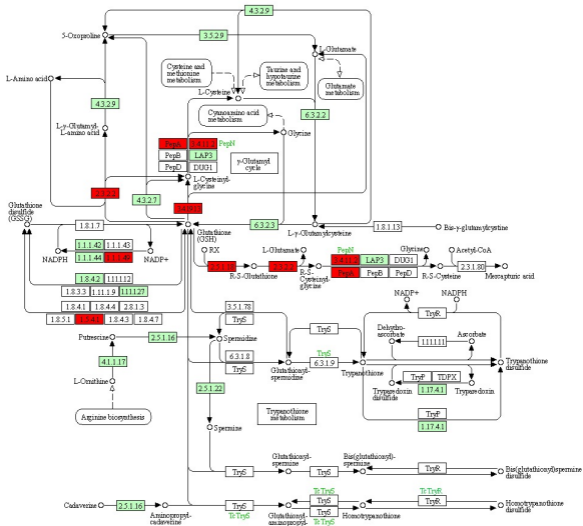

Supplement: Supplementary file 1 [file insects-13-00967-s001.zip › File S2.pdf]

## GLYCINE, SERINE AND THREONINE METABOLISM

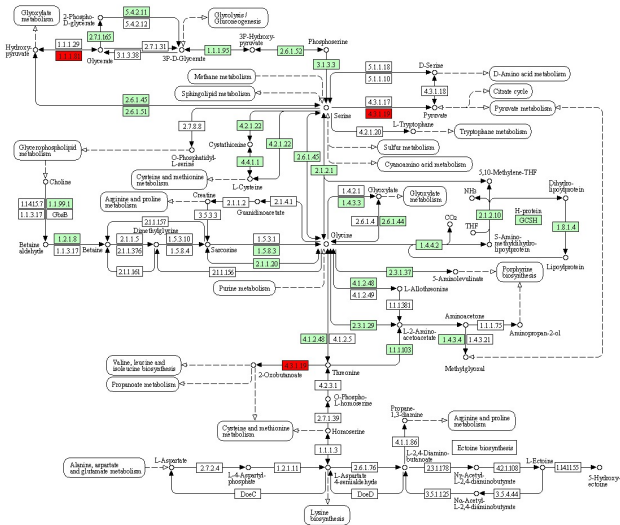

Supplement: Supplementary file 1 [file insects-13-00967-s001.zip › File S3.pdf]
